# The Optimization of Stand Structure Can Significantly Alleviate the Flammability of Forest Ecosystems

**DOI:** 10.1002/ece3.71343

**Published:** 2025-05-13

**Authors:** Yan Zhang, Xiangwen Deng, Xiaoyong He, Xiaolong Zhang, Zhihong Huang, Liang Chen, Shuai Ouyang, Wenhua Xiang

**Affiliations:** ^1^ College of Life and Environmental Sciences Central South University of Forestry and Technology Changsha Hunan Province China; ^2^ Huitong National Field Station for Scientific Observation and Research of Chinese Fir Plantation Ecosystem in Hunan Province Huitong China; ^3^ National Engineering Laboratory for Applied Technology of Forestry & Ecology in South China Changsha China; ^4^ Forestry Bureau of Shaoyang County Shaoyang China

**Keywords:** burning intensity & severity index, fire hazard, forest fuel, fuel treatment, ignition forest fire risk index, subtropical, vegetation

## Abstract

The accurate classification of forest fuels and the evaluation of the flammability of different forest types are crucial for effective forest fire control and classification management. We aimed to evaluate and classify the flammability of surface forest fuels in the subtropical area of China. The surface forest fuels were collected from 12 typical forest types. The flammability of surface forest fuels was assessed by evaluating their drying time, fuel moisture, ignition point, calorific value, combustion duration, and ash content. The principal component analysis (PCA), entropy weight method, k‐means clustering algorithm, and Pearson correlation coefficient method were employed for the classification of forest fuels and the evaluation of forest flammability. The results revealed that the flammability of surface living fuels across diverse plant families was significantly different. Rutaceae and Cucurbitaceae plants exhibited relatively high flammability, while Arecaceae plants demonstrated characteristics of low flammability. The surface fuels could be categorized into high, moderate, and low flammability. The high flammability fuels mainly consisted of plant leaves and litter components. The forest humus belongs to the low flammability. The forest flammability was classified into three categories according to the ignition forest fire risk index (IRI) and the burning intensity & severity index (BSI). The highest flammability forest types were EPF: *Pinus elliottii* pure forest, BMF: broad‐leaved mixed forest, CPF: *Cunninghamia lanceolata* (Lamb.) Hook pure forest, and CBF: coniferous broad‐leaved mixed forest. The lowest flammability was in FPF: *Liquidambar formosana* Hance pure forest, an optimal forest type with a neatly structured environment, few understory weeds, and less dead fuel loading of only 4.32 tons per hectare. The flammability index method presented in this study contains the key elements of flammability, provides a standardized tool for fire managers to assess and mitigate fire risk, and it also applies to other regions.

## Graphical abstract

1. Flammability of various plant families in surface living fuels varies significantly, and fuels of Rutaceae, Cucurbitaceae exhibited high flammability, while Arecaceae were low.
2. Leaves, litters were high flammability fuels, humus was low flammability fuel.
3. Flammability index including IRI & BSI can be used to classify stand combustibility, which showed that the fewer understory and less dead fuels, the lower flammability the forest type was.

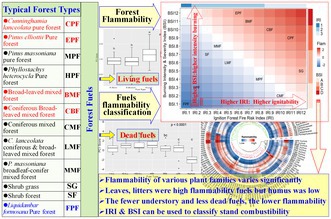

## 1 Introduction

Wildfires were increasingly frequent and severe due to global climate change, land‐use changes, and human activities near forests, causing large‐scale social, economic, and ecological damage (Cui et al. 2022; Pagadala et al. 2023; Zhao et al. 2024). The incidence of forest fires exerts a profound influence on ecosystem succession and plant community structure (Emery and Hart 2020; Pausas et al. 2017; Pausas and Keeley 2023), playing a paramount role in species distribution and landscape vegetation dynamics (Bond et al. 2004; Bond and Scott 2010; Pausas and Poorter 2014).

The occurrence and development of forest fires were influenced by various environmental factors, including forest fuels, ignition sources, and meteorological conditions (Rosavec et al. 2022). Forest fuels play an important role in starting and spreading, which serve as fundamental material for forest fires (Yang et al. 2021). Among the three components of fires, forest fuel stands out as the only element effectively manageable by humans, offering a key avenue for mitigating the adverse impacts of forest fires (Güney et al. 2022; Kreider et al. 2024; Yang et al. 2021). Plant flammability was influenced by diverse functional traits across species, communities, and vegetation levels (Iván et al. 2022). Exploring the correlation between plant traits and flammability is essential for understanding vegetation fire dynamics at both local and landscape scales in academic research (Babl et al. 2020; Popović et al. 2021). The composition and abundance of surface forest fuels exhibited significant variations among different forest types, resulting in corresponding discrepancies in their flammability (Li et al. 2023). Forest fire behavior was determined by the characteristics of these surface forest fuels (Anne et al. 2011; McColl‐Gausden and Penman 2019). The role of surface forest fuels is critical in both the onset and expansion of forest fires (Dong and Williams 2023; Neumann et al. 2022; Ying et al. 2020). The investigation into the combustibility of fuels facilitates the prediction of forest fire risk, which is a substantial aspect in managing forest ecosystems and establishing fire zones (Chiono et al. 2012).

The characterization of flammability as a material's ability to initiate and sustain combustion varies among authors and disciplines (Wyse et al. 2016). According to Anderson (1970) and Martin et al. (1994), fuel flammability was a multifaceted concept traditionally assessed through four components that encompass the ease of ignition (ignitability), the intensity of combustion (combustibility), the biomass of burning (consumability), and the duration of burning (sustainability). The flammability of ecosystems was determined by traits expressed at various biological and ecological levels, including the organ, species, population, and community (Cingolani et al. 2023; Pausas and Moreira 2012).

The flammability differences between plant species have been extensively investigated by numerous researchers employing various methodologies and testing criteria (Jian et al. 2024; Toy‐Opazo et al. 2024; Younes et al. 2024). Mutch's (1970) study proposed a species‐level approach to combustibles and their application at the community level, highlighting that surface dead fuels such as litter and fine wood debris serve as primary fire vectors in various vegetation types. The relationship between leaf traits, litter structure, and flammability of 106 plant species was analyzed by SEM in the study conducted by Burton et al. (2020). The flammability data of species were traditionally obtained through feature‐based studies, which commonly employ the mean values at the species level (Popović et al. 2021). It was found by Yue‐hong et al. (2018) that the moisture content and time lag were significantly affected by the type and distribution of fine combustible materials on the surface, with a positive correlation observed between the ratio of branches and these factors. It was revealed by Yan‐long et al. (2003) that there was a direct relationship between the content of plant extracts and the calorific value during combustion. Korená Hillayová et al. (2023) developed and tested a mathematical model for predicting wildfire risk in spruce stands within Slovak Paradise National Park under climate change conditions.

Furthermore, a portion of the spatial variation in surface dead fuel moisture of woodland and forest ecosystems was likely attributed to differences in stand conditions, such as stand density and composition (Kane 2021). The dead fuel moisture in dense stands with high canopy cover was typically higher compared to that in more open stands or grasslands (Tanskanen et al. 2006). The study conducted by Calitz et al. (2015) revealed that the flammability of alpine sous‐leaved shrubs and grasslands in the southern Cape of South Africa was attributed to their high density of slender branches. Selecting less flammable plant species is a recommended management strategy to reduce fire risk, but identifying them can be challenging given the complexity and dynamics of vegetation flammability (Kauf et al. 2014). However, the documentation of functional trait covariation consistency from individual to ecosystem levels remains limited (Alam et al. 2019; Pausas et al. 2017).

In this study, we collected 12 representative forest types in mid‐subtropical China to evaluate the flammability of forest by quantifying the combustibility of various surface forest fuels. The aims of this paper are to: (1) investigate the composition and quantities of surface forest fuels in the mid‐subtropical region of China, while analyzing their flammability characteristics, (2) categorize the surface forest fuels according to their flammability, and (3) assess the combustibility of various stand structures based on the ignition forest fire risk index (IRI) and burning intensity & severity index (BSI) of the surface forest fuels. We tested two hypotheses: (1) Variations in surface forest fuel flammability among mid‐subtropical forest ecosystems are influenced by differences in plant families, moisture, and fuel loading, and (2) This study aims to classify typical mid‐subtropical forest structures based on flammability, providing strategies for forest management to reduce combustibility. This issue is particularly prominent in China; however, it is also prevalent globally. Therefore, the findings of this study are not only significant for China but also offer valuable insights for other countries and regions. The research provided essential data that significantly contributes to mitigating forest fire risks through effective forest management, optimal fire prevention measures, and afforestation strategies.

## 2 Materials and Methods

### 2.1 Study Area

**FIGURE 1:**
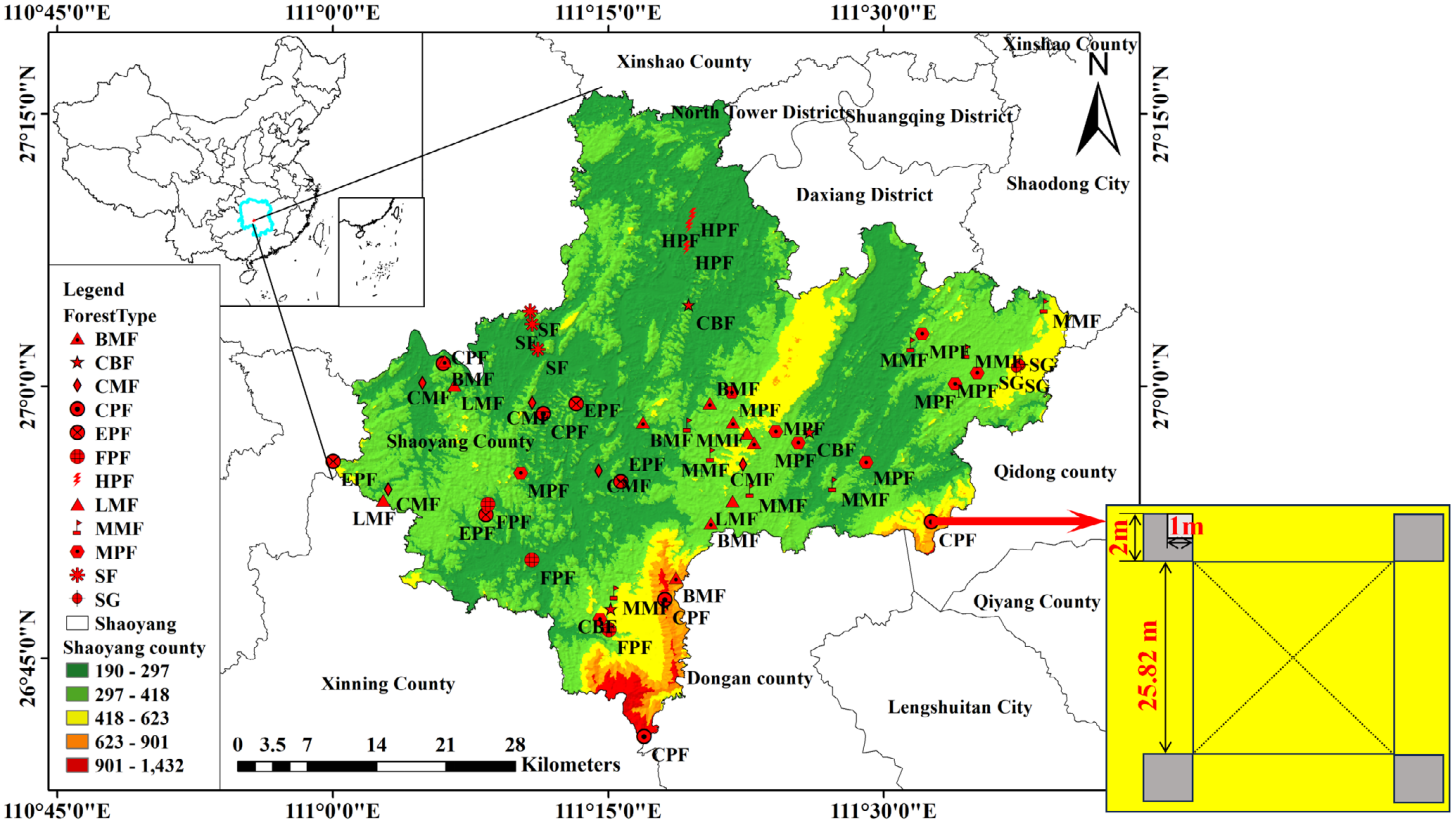
Study area and sample plot layout diagram. CPF: *Cunninghamia lanceolata* (Lamb.) hook pure forest, MPF: *Pinus massoniana* pure forest, EPF: *Pinus elliottii* pure forest, FPF: *Liquidambar formosana* Hance pure forest, HPF: *Phyllostachys heterocycla* (Carr.) Mitford cv. Pubescens pure forest, CMF: coniferous mixed forest, BMF: broad‐leaved mixed forest, CBF: coniferous broad‐leaved mixed forest, LMF: *Cunninghamia lanceolata* (Lamb.) hook coniferous and broad‐leaved mixed forest, MMF: *Pinus massoniana* broadleaf‐conifer mixed forest, SF: broad‐leaved shrub forest, SG: broad‐leaved shrub grass.

The sampling points are distributed in Shaoyang County, Hunan Province, in the mid‐subtropical region of China (26°40′36″‐27°16′18” N, 110°59′56″‐111°40′14″ E), at an elevation of 190–1450 m a.s.l. (Figure 1). The region is characterized by a subtropical monsoon humid climate. The forest types in the county were primarily characterized by pure coniferous forests, followed by broad‐leaved forests, mixed needle‐broadleaf forests, and shrub economic forests, which represent typical subtropical forest types (Ma et al. 2020; Wei et al. 2018).

### 2.2 Sample Plots

The sample plots were established in the selected 12 representative forest types within the mid‐subtropical region of China, each covering an area of 25.82 × 25.82 m. The research objects comprised a total of 104 sample plots, with each forest type having more than three sample plots. The shrub quadrat measuring 2 × 2 m and the herb quadrat measuring 1 *×* 1 m were established at the four corners of each sample plot. The diameter at breast height (DBH), tree height (H), and crown width of each tree species with a diameter exceeding 5 cm were meticulously measured. The comprehensive information of the stands is presented in Tables S1 and S2.

### 2.3 Fuel Loading Measurement and Sample Collection

The surface forest fuels were classified into two categories: live fuels, which encompassed shrubs (including young trees over 30 cm height), herbs (including young trees and shrubs below 30 cm height), and dead fuels (included dead fallen woods, litters, and humus). The total harvest method was employed to quantify the surface forest fuel loading (Xiang et al. 2023).

The above‐ground portions of all shrubs within the shrub quadrat were harvested and weighed. Three sample shrubs from the dominant species were selected to determine the proportion of their stems, branches, and leaves. The above‐ground portions of all herbs within the herb quadrat were also harvested and weighed. About 500 g of samples from each component, including shrub stems, branches, leaves, and herbs, were collected and transported back to the laboratory.

According to the diameter and delay, the litters were divided into three grades, named 1.h.L (1 h litters of diameter < 0.64 cm), 10.h.L (10 h litters of 0.64 cm ≤ diameter < 2.54 cm), 100.h.L (100 h litters of 2.54 cm ≤ diameter ≤ 7.62 cm) (Xiang et al. 2023). Those greater than 7.62 are classified as dead fallen wood. The dead fallen woods were categorized into half‐rotten wood (HW), fully rotten wood (RW) and unrotted wood (UW), and their loadings were measured in each stand. The fuel loadings of litters and humus were conducted within the herb quadrat. About 500 g of samples from each component, including HW, RW, UW, 1.h.L, 10.h.L, 100.h.L, and humus, were collected and transported back to the laboratory.

The surface forest fuel loading of each type was determined using the following formula:

(1) $$\mathrm{FL}=\left(D-M_{S}\right)/S/100$$

where FL represents the fuel loading (T/hm^2^), D is the dry‐fresh ratio of the sample, S is the sample area product (m^2^), 100 is the unit conversion coefficient, and M_s_ is the total fresh mass (kg) of fuel in the quadrate.

### 2.4 Measurement of Flammability Characteristics

All samples were dried in an oven at 85°C until they reached a constant weight, and their weights were measured every 10 min to document the water escape process. The total drying time (DT) of each sample was determined by calculating the recorded measurements in hours. The fuel moisture content (FMC) was calculated utilizing the following formula:

(2) $$\mathrm{FMC}=\left(D_{W1}-D_{W2}\right)/D_{W1}\cdot 100\%$$

where FMC represents the fuel moisture content, D_w1_ denotes the fresh weight of the sample, and D_w2_ is the dry weight.

The preheating energy consumption (PE) of all samples refers to the energy consumed before fire burning, and the formula is as follows:

(3) PE=FL·FMC/100

where PE is preheating energy consumption, FL is fuel loading of each forest type, FMC is fuel moisture of each forest type, and 100 is the unit conversion coefficient.

The ignition point (IP) refers to the minimum temperature necessary for initiating combustion of surface forest fuels. The combustion duration (CD) was quantified as the time interval from the ignition of surface forest fuels until the cessation of visible flames. The CD was recorded within seconds using the methodology outlined by Kane et al. (2021). The instrument employed in the combustion experiment was the microcomputer‐based ignition point tester (TRRD‐*2*A). Both ignition point (IP) and CD were obtained through the conducted combustion experiment.

The ash content (AC) of the sample was determined using the dry ashing method (Lixuan et al. 2022). The drying sample, weighing approximately 2 g, was transferred into a crucible and placed in a Muffle furnace. The crucible was gradually heated to 550°C and maintained at this temperature for 6 h for ashing. Then, it was cooled back down to room temperature and weighed. The AC was calculated using the following formula:

(4) $$\text{AC}=\left({\text{M}}_{1}-{\text{M}}_{0}\right)/\left({\text{M}}_{2}-{\text{M}}_{0}\right)\cdot 100\%$$

where AC represents the ash content of the surface forest fuels, M_1_ denotes the weight (g) of the sample after ashing in a crucible, M_2_ signifies the weight (g) of the crucible before drying and zoning the sample, and M_0_ refers to the net weight of the crucible itself.

The calorific value (CV) of the sample was determined using the Benzoic acid calibration calorimeter (HD‐C3000) combined with the oxygen bomb method.

### 2.5 Data Analysis

Shapiro–Wilk function and Bartlett test were used to test the normality and homogeneity of variance of the data. If the data did not conform to the normal distribution, the data should be logarithmically transformed.

The differences in flammability characteristics among different plant families were assessed using ANOVA. Principal component analysis (PCA) was conducted to research the flammability characteristics, utilizing the *princomp* function in a statistical package. First, we used ignition point (IP), combustion duration (CD), calorific value (CV), ash content (AC) to explore the flammability patterns of different plant families. We found that the first two axes explained nearly 83.5% of the variation in the data, and all flammability variables were positively correlated. Therefore, we used the scores of these four flammability variables on the first two axes to provide a flammability index for different plant families and for further exploratory analyses (see below). The higher the flammability index, the higher the flammability. This flammability index was further used to classify the studied taxa into three flammability levels as defined by Pausas et al. (2017): high, medium, and low. We applied K‐means clustering for flammability classification (Hartigan and Wong 1979). We conducted a classification analysis of all forest fuels using a consistent method (Figure 5). The second PCA (Figure S2) was conducted using the four flammability variables (ignition point (IP), combustion duration (CD), calorific value (CV), ash content (AC)) for different components of live and dead fuels. Considering that the first axis of the second PCA explained 84.3% of the variation in the data, we used the positions on this axis to perform ANOVA for different components of live and dead fuels separately, further exploring the differences in flammability among different components. To investigate the differences in fuel traits among taxa across various components, ANOVA analyses were conducted, followed by LSD tests. The relationship between flammability characteristics was investigated using Pearson correlation analysis. To study the flammability of different forest types, the IRI and BSI of different surface forest fuels were established by K‐Means clustering and entropy weight (Table 1). The data were analyzed using R 4.3.3 (Team 2024).

**TABLE 1:** Described the variable allocation of the ignition forest fire risk index (IRI) and the burning intensity & severity index (BSI).

Index	Variable
Ignition forest fire risk index (IRI)	Ignition point (IP)
	Fuel moisture (FM)
	Preheating energy consumption (PE)
	Fuel loading (FL)
	Drying time (DT)
Burning intensity & severity index (BSI)	Calorific value (CV)
	Ash content (AC)
	Fuel moisture (FM)
	Fuel loading (FL)
	Combustion duration (CD)

## 3 Results

### 3.1 Variation in Flammability and Surface Forest Fuels Traits

#### 3.1.1 Living Surface Forest Fuels

A total of 37 species of shrubs and 32 species of herbs were collected from the 12 forest types, belonging to 39 families and 52 genera. The flammability characteristics, including ignition point (IP), combustion duration (CD), calorific value (CV), ash content (AC), and DT, exhibited significant variations across the 69 surface living forest fuels (Table S3).

The analysis of variance (ANOVA) revealed a significant difference in the IP among different plant families. In the herb layers, the average IP of Theaceae was 244.8 ± 0.0 (°C), which was significantly higher than that of other plant families. Conversely, Primulaceae plants exhibited the lowest average IP with a value of 231 ± 5.10 (°C). Within the shrub layer, Cupressaceae plants displayed the highest average IP at 245.2 *±* 0.69 (°C), while Arecaceae plants had the lowest average IP at 220.8 ± 0.0 (°C).

The CD showed no significant variations among plant families in the herb layer. In contrast, notable disparities were observed in CD within the shrub layer. The Rutaceae plants exhibited the longest average CD of 65.3 ± 2.51 s, whereas Arecaceae plants displayed the shortest average CD of 53.8 ± 0.0 s.

The CV of the shrub layer and herb layer varied significantly across different plant families. Within the herb layer, Theaceae exhibited the highest average CV of 19.445 ± 0.0 (KJ/g); the Cucurbitaceae family demonstrated the lowest average CV at 16.236 ± 0.0 (KJ/g). In the shrub layer, Ericaceae exhibited the highest CV at 18.746 ± 0.63 (KJ/g), whereas the Arecaceae family demonstrated the lowest CV at 16.375 ± 0.0 (KJ/g).

The AC exhibited significant variations across plant families. In the herb layer, the Moraceae exhibited the highest average AC, reaching up to 10.7 ± 0.0 (%), while Theaceae had an average AC as low as 3.5% ± 1.3 (%). Within the shrub layer, Verbenaceae displayed the highest average AC (5.8% ± 1.3%), while Arecaceae exhibited the lowest (2.6% ± 0.0%), representing the minimum observed value.

Significant differences in DT were observed among different plant families. The herb layer exhibited the longest average DT of 55.8 ± 0.0 h for Celastraceae, while the Cucurbitaceae family had the shortest DT of 24.0 ± 0.0 h. The Arecaceae family exhibited the most significant DT (147.8 ± 10.8 h) among the shrub layer, while the Elaeagnaceae family had the shortest average DT (33.6 ± 0.0 h).

To summarize, significant differences in flammability characteristics, such as the IP, CD, CV, AC, and DT were observed among plant families in the herb and shrub layers. The IP and CV of plants belonging to Theaceae in the herb layer exhibited the highest values, whereas those of plants from Arecaceae in the shrub layer displayed the lowest IP and shortest CD but the longest DT (Table S3).

**FIGURE 2:**
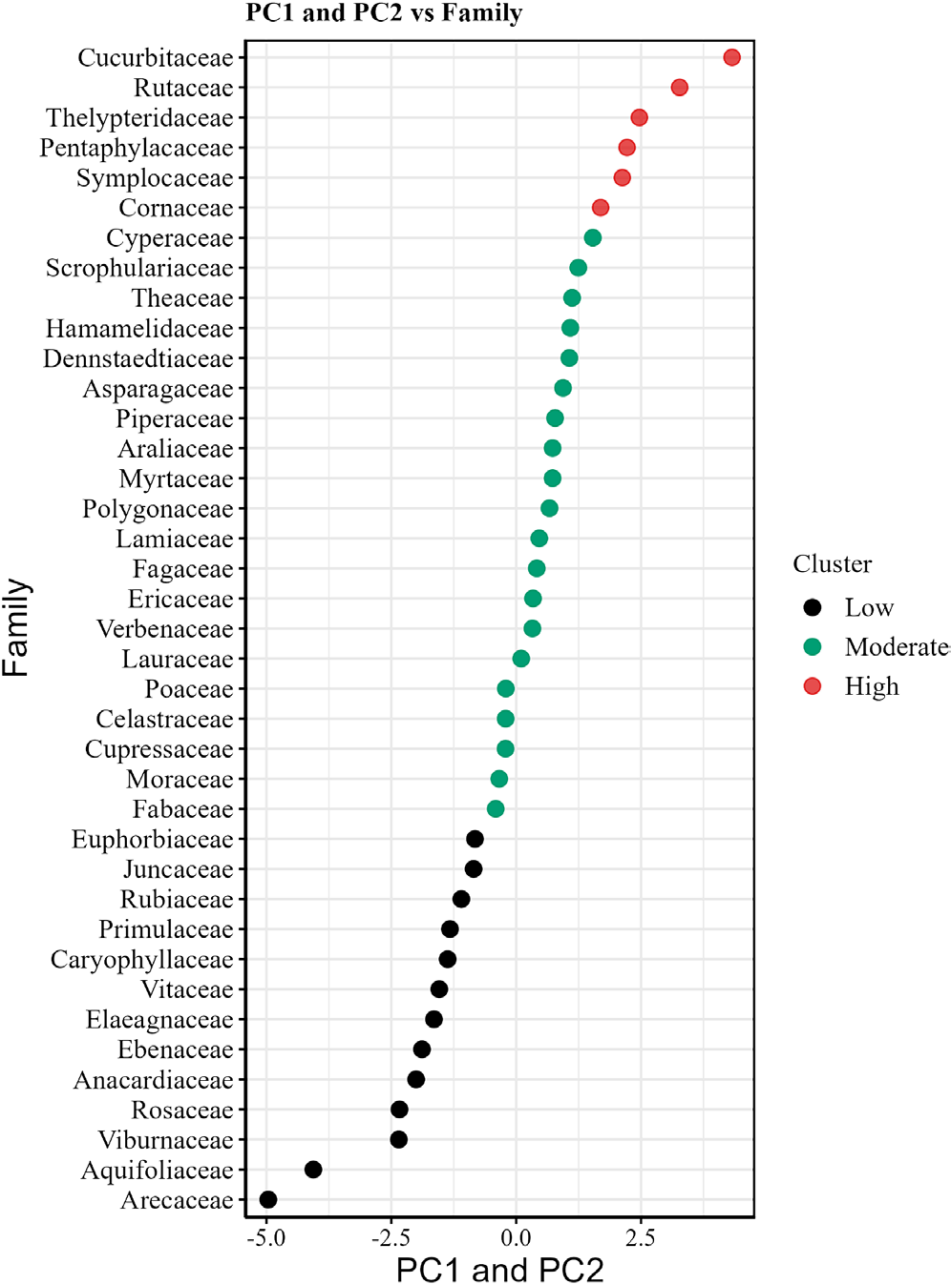
The flammability ranked and clustered across the 39 different families by PC1 and PC2 scores.

The first two axes explained 83.5% of the total variation in flammability data. The first axis of PCA conducted with the flammability variables explained 44.7% of the variation of the data in the flammability of families and exhibited positive associations with all four flammability traits: IP (loading = 0.41), CV (loading = 0.56), CD (loading = 0.82), and AC (loading = 0.86) (Figure 2, Figure S1). The second axis (PC*2*) explains 38.8% of the variation in flammability. It was positively correlated with IP (loading = 0.80) and CD (loading = 0.69) while being negatively correlated with CV (loading = − 0.39) and AC (loading = − 0.46). Notable variations in flammability were observed among taxonomic families (Figure 2). Cucurbitaceae and Rutaceae exhibit the highest level of flammability, followed by Thelypteridaceae and Pentaphylacaceae. The Arecaceae and Aquifoliaceae display the lowest degree of flammability, followed by Viburnaceae and Rosaceae. The flammability of taxa from other families was at a moderate level (Figure 2).

**FIGURE 3:**
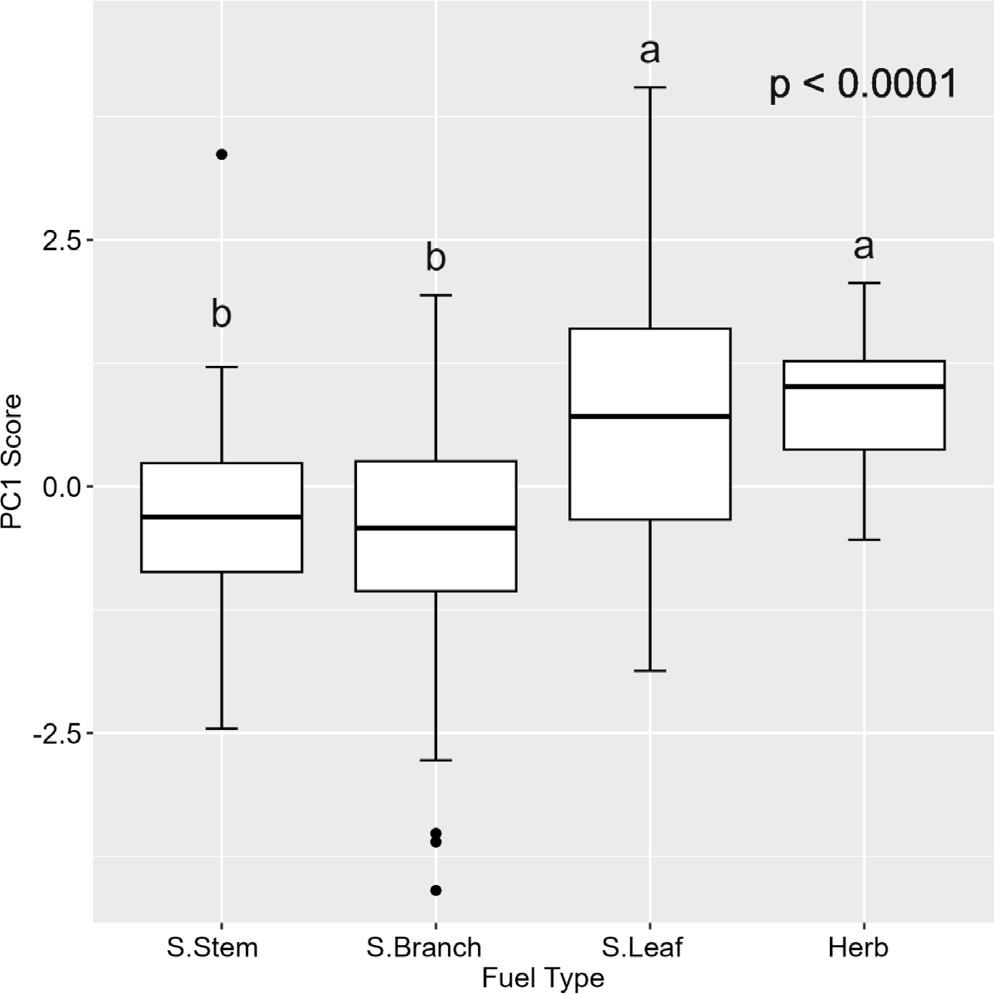
Boxplots visualizing the variation in flammability (PC1 score) of taxa between different live fuel components (stem, branch, and leaf of shrub and herb). Different letters denote significant differences among groups (*p* < 0.0001). S.Stem: The stem of the shrub, S.Branch: The branch of the shrub, S.Leaf: The leaf of the shrub.

The second PCA was performed using four flammability variables (IP, CV, AC, and CD) of different components of different forest fuels (Figure S2). The first two axes of principal component analysis accounted for 98.7% of the total variance, among which the first principal component axis accounted for 87.5% of the data variance and was positively correlated with IP (loading = 0.95), AC (loading = 0.96) and CD (loading = 0.91). It is negatively correlated with CV (loading = − 0.91). The second axis of PCA accounted for 11.5% of the total variation and was positively correlated with IP (loading = 0.27), CV (loading = 0.40) and CD (loading = 0.40). It was negatively correlated with AC (loading = − 0.26) (Figure S2). This first axis can be utilized to perform an analysis of variance (ANOVA) for the different components of different forest fuels (Figure 3). The PC1 score reveals significant variations in flammability among different components of surface live fuels (Figure 3). Leaves demonstrate the highest level of flammability, followed by herbs and stems, while branches have relatively low flammability.

#### 3.1.2 Dead Surface Forest Fuels

**FIGURE 4:**
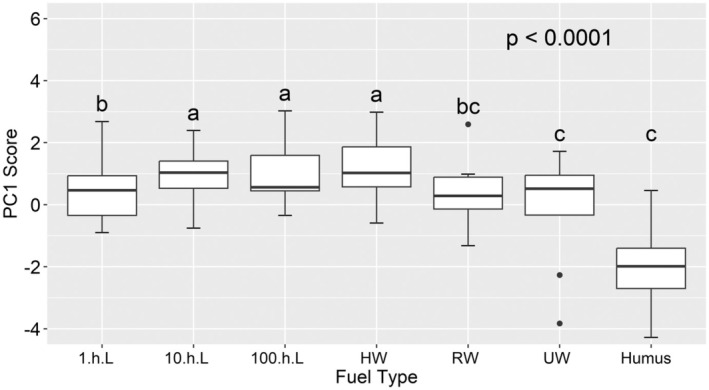
Boxplots visualizing the variation in flammability (PC1 score) of taxa between different dead fuel components. Different letters denote significant differences among groups (*p* < 0.0001). 1.h.L is the 1 h litters, 10.h.L is the 10 h litters, 100.h.L is the 100 h litters, HW is the half rotting wood, RW is the rotten wood, UW is the unrotted wood.

The PC*1* scores in the second PCA (Figure S2) also revealed the flammability relationship among different components of surface dead fuels. The variance analysis of surface dead fuels reveals significant variations in flammability (Figure 4). Humus exhibited relatively low flammability, followed by UW and 1.h.L, while the most flammable components are those of 10.h.L, 100.h.L, and HW (Figure 4).

### 3.2 Flammability Classification of Surface Forest Fuels

To identify and exclude indicators with strong collinearity using VIF (variance inflation factor), five indicators were selected for cluster analysis based on this analysis. The K‐means analysis categorizes different types of forest fuels into three levels of flammability: high, moderate, and low flammability.

**FIGURE 5:**
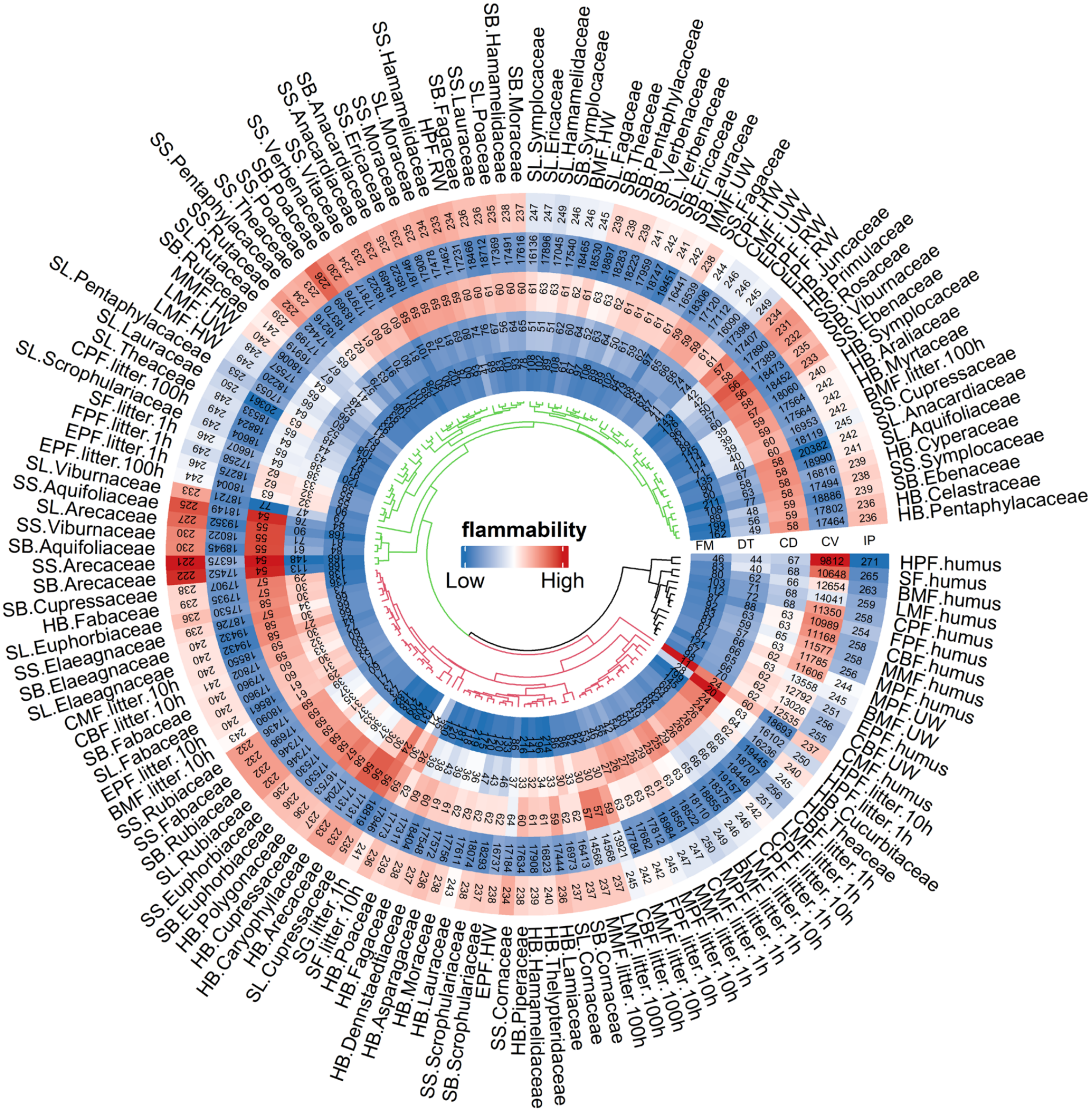
Presents a comprehensive ring heat map of cluster analysis for diverse components in different surface forest fuels. In the inner ring cluster, black represents a low flammability class, red represents a high flammability class, and green represents a medium flammability class. SS: Stem, SB: Branch, SL: Leaf, HB: Herb, HW is the half‐rotting wood, RW is the rotten wood, UW is the unrotted wood, IP (°C): Ignition point, CD (s): Combustion duration, CV (KJ/g): Calorific value, DT (h): Drying time, FM (%): Fuel moisture. The forest types abbreviations are shown in Figure 1.

The K‐means clustering highlights low flammability components such as humus. Humus of different forest types exhibits similar flammability characteristics, typically featuring a high IP, low CV, extended DT, and relatively prolonged CD (Figure 5). The high flammability components consisted of dead surface forest fuels, including 1.h.L and 10.h.L in all types of forests, as well as live surface forest fuel comprising branches and leaves from various plant families. The high flammability components exhibited common characteristics including a low IP, a short required DT, a relatively short overall CD, and a low FM (Figure 5).

### 3.3 The Flammability Characteristics of Different Forest Types

#### 3.3.1 Fuel Loading of Different Forest Types

**FIGURE 6:**
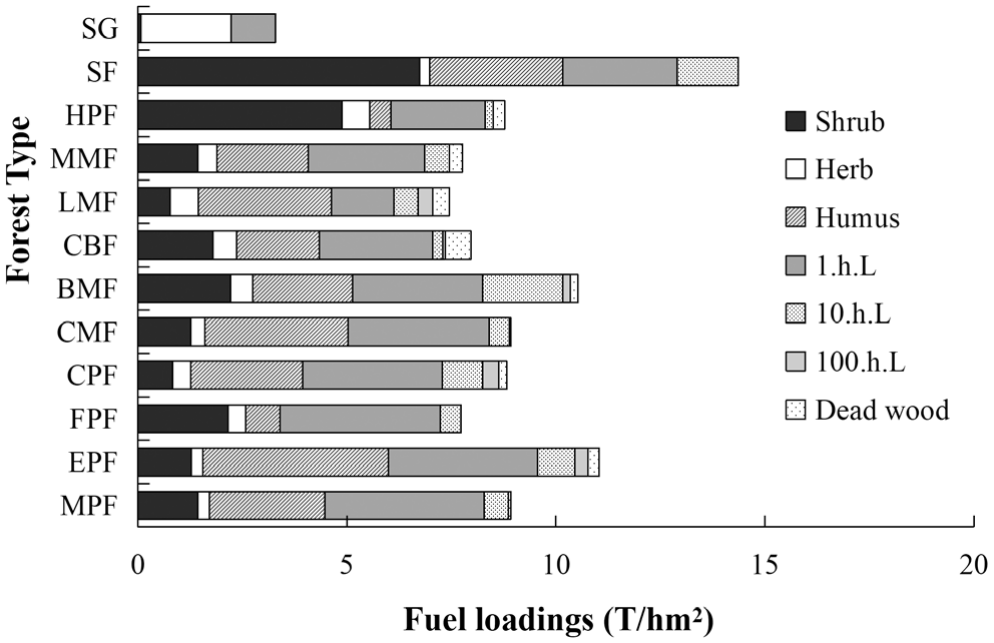
Fuel loading of different components in 12 forest types. Abbreviations of forest types are the same as in Figure 1.

The fuel loading varied significantly among different forest types in terms of the fuel components (Figure 6). The SF exhibited the highest surface fuel loading in the shrub layer, with a value up to 6.74 (T/hm^2^), while SG, LMF, and CPF had relatively lower surface fuel loading. Herb fuels from SG contributed significantly to the overall surface fuel loading, whereas SF had the lowest herb surface fuel loading. BMF contained a higher surface fuel loading of litter compared to other forest types. The humus fuel loading in HPF and FPF was relatively lower compared to other forest types. Considerable variations and skewed distributions were observed in most of the fuel components, especially in shrub and herb fuels (Figure 6).

#### 3.3.2 Relationship Between Flammability Variables and Fuel Traits

**FIGURE 7:**
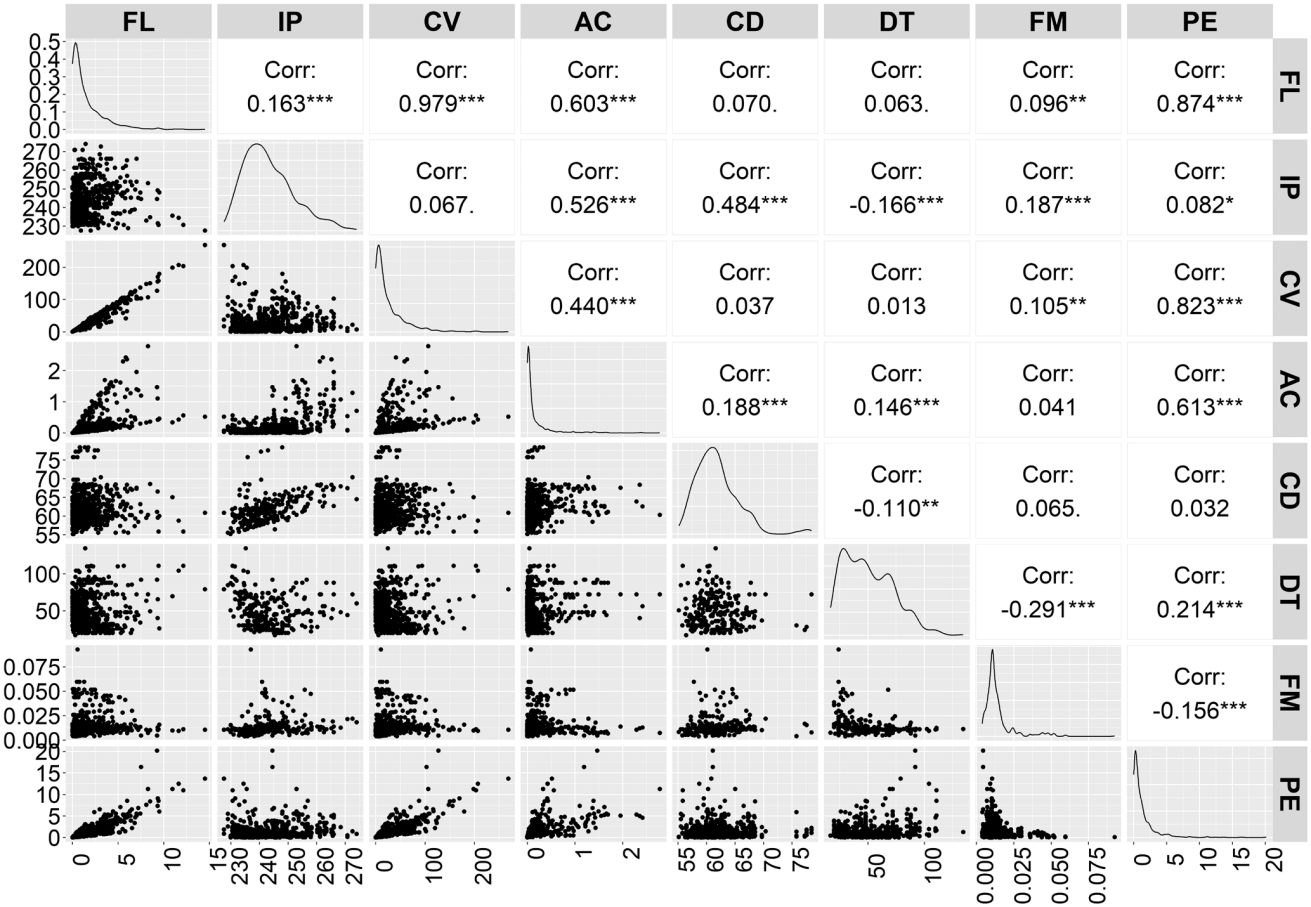
Correlation matrix visualizing the Pearson's correlation coefficient between the flammability variables (IP: Ignition point, CV: Calorific value, AC: Ash content, CD: Combustion duration, PE: Preheating energy consumption), fuel traits (FL: Fuel loading, DT: Drying time, FM: Fuel moisture). Statistically significant correlations are marked by an asterisk. *, **, *** Significant at *p* ≤ 0.05, 0.01, and 0.001, respectively.

The correlation matrix revealed significant correlations among the flammability variables and fuel traits (Figure 7). There was a significant positive correlation between FL and IP, CV, AC, and PE, as well as a significant negative correlation between FL and FM The IP was positively correlated with AC and CD, negatively correlated with DT and FM, and slightly positively correlated with PE. The CV was highly significantly positively correlated with AC and PE, and negatively correlated with FM. The AC was highly significantly positively correlated with CD, DT, and PE, but was nonsignificantly negatively correlated with FM. There was a highly significant positive correlation between PE and FM (Figure 7).

#### 3.3.3 Flammability Classification of Forest Types

**FIGURE 8:**
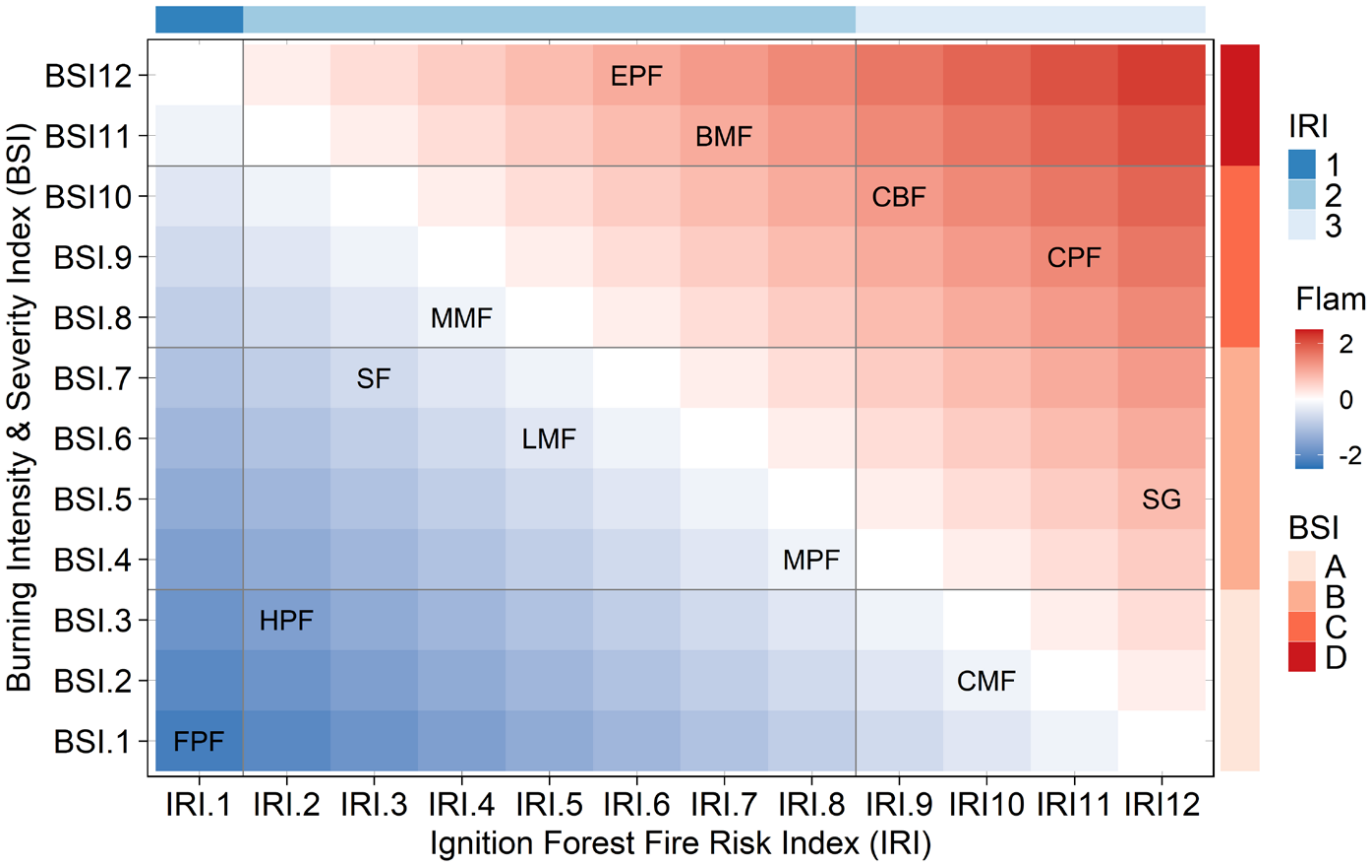
Heat maps displaying the ignition forest fire risk index (IRI) and burning intensity & severity index (BSI) for the 12 forest types, along with the respective cluster results. See Figure 1 for the abbreviations of the 12 forest types.

The 12 forest types were classified into three categories, with labels 1, 2, and 3, according to the IRI. The SG, CPF, CMF, and CBF belonged to the highest ignitability categories, followed by MPF, BMF, EPF, LMF, MMF, SF, and HPF, which belonged to medium ignitability categories. The FPF was the lowest ignitability category (Figure 8).

According to BSI, the 12 forest types were classified into four categories, with labels A, B, C, and D. The EPF, BMF were the highest intensity burning categories, followed by CBF, CPF, MMF as the intense burning categories. The SF, LMF, SG, and MPF were the medium intensity burning categories. The HPF, CMF, and FPF were the low intensity burning categories (Figure 8).

There were significant differences in the flammability of the 12 forest types based on the combined score for IRI and BSI. The labels A1 and D3 denoted the stands with the lowest and highest flammability, respectively. The EPF and BMF, labeled as D2, along with CBF and CPF, labeled as C*3*, exhibited a relatively high level of flammability. Additionally, SG, labeled as B3, also demonstrated a similar characteristic, which is characterized by medium‐high ignitability and high‐intensity burning. The FPF, labeled as A*1*, belonged to the lowest level of flammability, and demonstrated the lowest ignitability and low‐intensity burning, followed by HPF, labeled as A2 (Figure 8).

## 4 Discussion

### 4.1 Flammability of Surface Forest Fuels

The study conducted by McAllister and Weise (2017) revealed significant disparities in flammability characteristics between live and dead plant material, highlighting the crucial role of interspecies variability. We found that there were significant differences in the flammability characteristics among different plant families (Figure 2). The taxa of Cucurbitaceae and Rutaceae demonstrated a potentially higher level of combustibility, possibly due to the presence of flammable compounds or structures in these plants that increase their susceptibility to ignition when exposed to fire or a burning source. In the management of forest ecosystems with a significant presence of these plants, it is essential to consider their potential fire risks and implement additional fire prevention measures or monitoring methods to mitigate potential fire hazards. In contrast, plants belonging to the Arecaceae family exhibit a lower flammability, which can be attributed to their unique biochemical composition or growth characteristics, such as higher DT and AC, low CV, robust leaves and stems, and a wax layer and fibrous tissue. Therefore, in the context of forest ecosystem management, these low‐burning plants can be effectively employed in the construction of biological fire‐prevention projects and ecological firebreak belts. Potts et al. (2022) discovered that legumes and camphoraceae exhibit notably high flammability, whereas Moraceae demonstrate significantly low flammability. However, our study indicates that legumes, Camphoraceae, and Moraceae are classified as moderately combustible. In addition, according to Cui et al. (2023) and Pagadala et al. (2023) both Rosaceae and Rhododendraceae exhibit high flammability. Conversely, our study reveals that Rosaceae may possess greater resistance to burning, potentially attributed to their higher AC, DT, and lower CV. The observed differences may be influenced by factors such as plant characteristics, growth conditions, and the design of combustion experiments, such as the selection of flammability variables. Our finding highlights the intricate and diverse nature of studying plant combustion characteristics, as well as the variations in flammability exhibited by plant families across various ecosystems and geographical locations. Further research could include more extensive sampling of different plant taxa, multidimensional assessment of combustion properties, and more refined experimental designs to delve deeper into the variability and factors influencing plant combustion properties.

The flammability order of different live combustibles was as follows: leaf > herb > branch > stem (Figure 3), which was consistent with the findings of Alam et al. (2019) study. Their research demonstrated that leaf‐level investigations were commonly conducted in fire ecology and fire evolution studies due to their feasibility, as leaves were typically the plant component that ignites first and is presumed to drive flammability. The flammability of plants can be increased by the presence of certain volatile organic compounds (VOCs), such as tannins, terpenes, isoprenoids, and phenolics (Page et al. 2012). Burton et al. (2020) found that leaves have both direct and indirect effects on the flammability of fuel beds. The findings of Stevens et al. (2020) suggested that in the forest ecosystems under investigation, litter flammability typically serves as the primary fuel source, while living forest fuels from shrubs and trees exert a more pronounced influence on fire behavior. Genetic factors and ecological environment contribute to significant variations in the same tree species. Additionally, the combustibility of a particular tree species varies at different growth stages (Pengtao et al. 2022). Hence, the combustibility of plants or tree species is directly or indirectly influenced by their biological and ecological characteristics (Zanzarini et al. 2022).

The combustibility of various dead forest fuels revealed the following sequence: litter > dead wood > humus (Figure 4). These findings align with (Xiang et al. 2023) in the eastern Tianshan Mountains of Xinjiang. The combustion of litter, often the primary fuel for fire propagation in forest fires (Lixuan et al. 2022), releases a substantial amount of energy, thereby facilitating fire expansion. The moisture content of dead fuels is a critical determinant of forest fire behavior and essential for accurate fire prediction and modeling (Ostrihoň et al. 2024). The moisture content of humus was typically higher, rendering it susceptible to decomposition by microorganisms and thus exhibiting the lowest relative flammability. Consequently, humus does not undergo direct combustibility during a fire, but humus serves as the primary fuel for subterranean forest fires (Heng et al. 2020).

### 4.2 Flammability Classification of Different Fuels

In our study, we found that different types of fuels classified into the same category exhibited similar combustion characteristics (Figure S3). The humus of each forest type was classified within the low flammability due to its biochemical characteristics, including higher IP, lower CV, prolonged DT, and relatively extended CD (Figure 5). These observations align with previous studies conducted by Zhigao et al. (2022) and Xiang et al. (2023), which also reported higher IP and lower CV for humus from various species.

The surface forest fuels, including 1.h.L and 10.h.L fuels, as well as branches and leaves from all the plant families, were classified into high flammability. The highly flammable category exhibited relatively low FM, which was similar to the findings reported by Dehane et al. (2017), highlighting flammable shrub species such as *Calicotome spinosa* and *Pinus halepensis* ; these species possess lower FM compared to other species.

We concluded that herb plants could be classified as having a moderate level of flammability. The results of our study, however, did not precisely align with the findings of Pagadala et al. (2023), which indicated a low flammability for the majority of herbs in agricultural landscapes. This suggested that there was minimal fire risk within vegetation communities. This discrepancy may be attributed to the fact that our herb samples were sourced from forest communities. The study conducted by Pausas et al. (2017) revealed that grass exhibited high susceptibility to ignition and rapid spread, yet demonstrated low combustion temperature and intensity, aligning with our own findings.

### 4.3 Comprehensive Flammability of Different Forest Types

The characteristics of forest fire behaviors, such as fire intensity, energy release, and burning rate, varied across different forest types due to the heterogeneous classification of surface forest fuels (Tian and Xueying 2013; Wang et al. 2024). In this study, we found high variability in fuel loading across various forest types and combustible components (Figure 6). Understanding the drivers of this variation will help us to quantify the spatial distribution of fuel loading. Our study found that dead forest fuels are a major component of fuel loading, the same as demonstrated by Ascoli et al. (2020). The substantial accumulation of dead forest fuels plays a crucial role in determining the combustibility of forest ecosystems by influencing their overall burning duration time (Fengjun et al. 2016).

The flammability of the forest ecosystem exhibited significant correlations with all fuel traits measured in this study (Figure 7). There was a notable negative correlation observed between FM and flammability. The forest types with a higher number of plant families such as Arecaceae and a greater amount of humus exhibited higher FM values, resulting in low flammability. The majority of studies have consistently demonstrated that higher fuel moisture content (FMC) in leaves, twigs, terminal branches, litter, wood, and bark was associated with a reduced ability of biomass to ignite (Bianchi et al. 2019; Molina et al. 2018; Scarff et al. 2021). It was also demonstrated that species with faster moisture loss rates exhibited higher flammability by Pagadala et al. (2023) and Cubino et al. (2018). Few studies have reported no significant effect of plant material's FMC on ignition (Popović et al. 2021).

When studying the impact of different species on flammability, most studies primarily focus on fuel beds consisting of a single species. Limited attention has been given to investigating the influence of two or more species on flammability (Varner et al. 2015). The species richness in forest ecosystems contributes to litter layer accumulation and the complex surface forest fuels (Magalhães and Schwilk 2012). The complex surface forest fuels of forest ecosystems were different from those of a single species during combustion, resulting in significant diversity of flammability variations, such as changes in flame size or prolongation of duration (Cassandra et al. 2012). The integration of species flammability scores with weighted community flammability scores has been employed in several studies to comprehensively assess different forest ecosystems, contributing to a better understanding of forest ecosystem flammability (Stevens et al. 2020).

To better quantify the flammability of forest ecosystems and effectively classify them, we had selected two indices, IRI which reflected the level of ignition difficulty and BSI which reflected the degree of combustion intensity, respectively (Madrigal et al. 2011; White and Zipperer 2010). In accordance with the IRI, the SG, CPF, CMF, and CBF exhibited the highest ignitability, which could be attributed to the fact that 1.h.L with lower FM and higher IP constitutes a significant proportion of the overall FL (Figures 5 and 6). The FPF had the lowest ignitability (Figure 8), which may be due to the fact that the FPF was a broadleaf pure forest with a higher nutrient return rate of litter (Nonghuloo et al. 2020; Zhang et al. 2019) and FM in the surface forest fuels, which was not easy to ignite. We categorized the forest types based on BSI, among which EPF, BMF belonged to the highest burning intensity, which may be due to the fact that the total amount of FL of these two forest types was relatively higher, which made the combustion duration longer. The HPF, CMF, and FPF were in the lower burning intensity category (Figure 8), which had a relatively low overall FL capacity and a short combustion duration.

The study conducted by Potts et al. (2022) emphasized the significant influence of different vegetation types on fire risk. The occurrence of fires was most likely in forest types characterized by low ignition temperatures, lower fuel moisture, higher accumulation of dead materials, and low moisture content (Pagadala et al. 2023). The EPF, BMF, CBF, and CPF, labeled as D*2* and C*3*, exhibited relatively high flammability characterized by the highest ignitability and the highest burning intensity. The forest type of EPF we selected was a mature pure coniferous forest, characterized by a consistently high understory accumulation of FL (6.33 T/hm^2^). Here, the BMF represented a typical example of a low‐quality and low‐efficiency broadleaf forest with a relatively low tree density (800 trees per hectare) in southern China. It exhibited a limited degree of canopy closure (below 0.5), resulting in abundant understory biomass (7.95 T/hm^2^). Therefore, it was recommended to increase the planting of arboricultural species. The situation in the forests of CBF and CPF was similar, and the understory accumulated a high level of surface forest fuels. While FPF labeled as A*1* had the lowest flammability (Figure 8) because of their moderate stand density (800 trees per hectare) and higher degree of canopy closure (upon 0.5) (Table S1), which resulted the lower understory herbs and lower shrubs fuel loading (2.58 T/hm^2^) (Figure 6), lower accumulation of total litters (4.32 T/hm^2^), so the IRI and BSI were in lower levels. Also, in HPF, the accumulation of litter was also lower (2.45 T/hm^2^), with lower levels of IRI and BSI. The remaining SG, MMF, SF, LMF, MPF, and CMF were of medium flammability, among which SG showed low—medium flammability, which was similar to the results of Yunjie et al.'s (2024) study, in which the weak combustibility of the shrub grass was relatively low fire hazard. The study conducted by Calitz et al. (2015) revealed that shrub grass communities exhibited a low level of flammability.

To effectively prevent forest fires, it was imperative to enhance the management of surface forest fuels both in high flammability and moderate flammability forest types (Zhao et al. 2024). Our findings indicate that pure coniferous forest types exhibited a high flammability to fire, incorporating certain broad‐leaved species could potentially alleviate the surface fire risk associated. The aforementioned perspective was bolstered by additional research that underscores the potential positive impacts of mixed forest (Anne et al. 2011; Cingolani et al. 2023; Schwilk and Caprio 2011). Moreover, studies have shown that in other regions with conditions different from our study site, using the cost‐effective and effective method we employed can yield similar flammability ranking results. Therefore, the method we describe can also be applied to other agricultural regions worldwide, and the results are similarly applicable to other similar geographical environments or ecological contexts (Murray et al. 2023; Pagadala et al. 2023; Zanzarini et al. 2022). These findings bear significant practical implications for the management of fire risks and the protection of ecosystems. To confirm these findings more strongly, it is imperative to conduct more extensive experimental investigations, particularly within undisturbed natural habitats.

Although this study emphasized the importance of applying flammability variable analysis in forest fire risk assessment, it also revealed some limitations. For instance, the restricted number of plots and their spatial clustering constrained the analysis of the flammability of these forest types. Future studies should focus on currently under‐represented forest communities to obtain fuel information with comparable precision. Additionally, incorporating more comprehensive variables of fire propagation rates, as well as other factors such as climatic conditions and topography, would enhance the understanding of vegetation flammability.

## 5 Conclusion

Our findings emphasized the potential influence of surface forest fuels and different forest types on fire behavior. The classification and ranking of the flammability characteristics of forest types revealed that conifer pure forests generally exhibited higher flammability than mixed conifer‐broad forests and broadleaf pure forests. The optimal forest management objective is to achieve a stand structure characterized by moderate stand density and high stand cover, such as that of *Liquidambar formosana* pure forest (FPF), which demonstrated full forest functionality and lower flammability. Research spanning from individual trees to forest ecosystems underscores the necessity for appropriate forest management measures to mitigate potential fire severity.

## Author Contributions

**Yan Zhang:** conceptualization (lead), data curation (equal), formal analysis (equal), methodology (equal), visualization (lead), writing – original draft (lead). **Xiangwen Deng:** conceptualization (lead), data curation (equal), funding acquisition (equal), investigation (equal), supervision (equal), writing – review and editing (lead). **Xiaoyong He:** investigation (equal), resources (equal), writing – review and editing (equal). **Xiaolong Zhang:** investigation (equal), resources (equal), writing – review and editing (equal). **Zhihong Huang:** methodology (equal), writing – review and editing (equal). **Liang Chen:** investigation (equal), writing – review and editing (equal). **Shuai Ouyang:** data curation (equal), investigation (equal), writing – review and editing (equal). **Wenhua Xiang:** funding acquisition (equal), writing – review and editing (equal).

## Conflicts of Interest

The authors declare no conflicts of interest.

## Supporting information


Data S1.

## Data Availability

The data that supports the findings of this study are available in the Supporting Information of this article.
