# Supplementary material for: The Optimization of Stand Structure Can Significantly Alleviate the Flammability of Forest Ecosystems
Source: Ecol Evol. 2025 May 13;15(5):e71343. doi: 10.1002/ece3.71343 (PMC12074718; doi:10.1002/ece3.71343)
Supplement: Supplementary file 1 — Data S1. [file ECE3-15-e71343-s001.docx]

**Supplementary materials:**

**Supplementary Tables**

**Table S1**. Forest type description.

| Forest Type | Code | H/m | DBH/cm | Altitude / m | Soil type | DCC/% | Den |
| --- | --- | --- | --- | --- | --- | --- | --- |
|  |  |  |  |  |  |  |  |
| *Cunninghamia lanceolata* (Lamb.) Hook pure forest | CPF | 10.74 | 18.1 | 658.97 | Yellow soil | 0.68 | 2633.32 |
| *Pinus massoniana* pure forest | MPF | 10.25 | 18.3 | 330.85 | Yellow soil | 0.69 | 1482.35 |
| *Pinus elliottii* Pure forest | EPF | 10.38 | 18.4 | 388.54 | Yellow soil | 0.68 | 1225.71 |
| *Liquidambar formosana* Hance Pure forest | FPF | 11.87 | 21.2 | 268.73 | Red soil | 0.75 | 760.00 |
| Coniferous mixed forest | CMF | 9.49 | 15.73 | 312.50 | Yellow soil | 0.57 | 1331.99 |
| Broad-leaved mixed forest | BMF | 11.20 | 19.2 | 391.84 | Yellow soil | 0.75 | 1259.99 |
| *Coniferou*s Broad-leaved mixed forest | CBF | 9.86 | 17.8 | 280.22 | Yellow soil | 0.74 | 1259.99 |
| *Cunninghamia lanceolata* (Lamb.) Hook coniferous and broad-leaved mixed forest | LMF | 11.10 | 18.8 | 352.19 | Yellow soil | 0.77 | 1031.99 |
| *Pinus massoniana* broadleaf-conifer mixed forest | MMF | 10.63 | 19.1 | 319.36 | Yellow soil | 0.71 | 1607.99 |
| *Phyllostachys heterocycla* (Carr.) Mitford cv. *Pubescens* Pure forest | HPF | 10.49 | 13.3 | 216.00 | Yellow soil | 0.85 | 3959.98 |
| Broad-leaved shrub forest | SF | 4.22 | 2.6 | 292.60 | Lithomorphic soil | 0.93 | 18933.33 |
| Broad-leaved shrub grass | SG | 1.02 | 1.2 | 427.60 | Lithomorphic soil | 0.05 | 533.33 |

**Note:** DBH: Average DBH. H: Average tree height. Den: Density. DCC: Degree of canopy closure.

**Table S2**. Basic Information Table of Various Vegetation Types in Different Regions.

| Forest Type | Plot-n | DBH | H | Den | DCC | Age | Arborous layer | Shrub layer | Herbaceous layer |
| --- | --- | --- | --- | --- | --- | --- | --- | --- | --- |
| HPF | HPF-1 | 13.33 | 10.47 | 3960 | 1.00 | 10 | *Phyllostachys edulis* | *Camellia oleifera* | *Pteridium aquilinum* |
| HPF | HPF-2 | 13.54 | 10.71 | 4680 | 1.00 | 10 | *Phyllostachys edulis* | *Camellia oleifera* | *Vitex negundo* |
| HPF | HPF-3 | 13.07 | 10.30 | 3240 | 0.93 | 10 | *Phyllostachys edulis* | *Various species of scattered bamboo* | *Trachycarpus fortunei* |
| CBF | CBF-4 | 16.13 | 10.04 | 1260 | 1.00 | 20 | *Melia azedarach* | Other shrubs | *Juncus effusus* |
|  |  |  |  |  |  |  | *Pinus elliottii* |  |  |
|  |  |  |  |  |  |  | *Cunninghamia lanceola* |  |  |
| CBF | CBF-5 | 13.77 | 9.00 | 840 | 0.90 | 20 | *Cupressus funebris* | *Cunninghamia lanceolata* | *Cunninghamia lanceolata* |
|  |  |  |  |  |  |  | *Liquidambar formosana* |  |  |
|  |  |  |  |  |  |  | *Melia azedarach* |  |  |
| CBF | CBF-6 | 15.97 | 9.49 | 1080 | 1.00 | 20 | *Cinnamomum camphora* | Other shrubs | *Juncus effusus* |
|  |  |  |  |  |  |  | *Pinus elliottii* |  |  |
|  |  |  |  |  |  |  | *Cupressus funebris* |  |  |
| MMF | MMF-7 | 26.56 | 12.57 | 1260 | 0.87 | 20 | *Paulownia fortunei* | Other shrubs | *Persicaria viscosa* |
|  |  |  |  |  |  |  | *Pinus massoniana* |  |  |
|  |  |  |  |  |  |  | *Phyllostachys edulis* |  |  |
| MMF | MMF-8 | 14.92 | 8.92 | 1920 | 1.00 | 20 | *Pinus massoniana* | *Triadica sebifera* | *Miscanthus sinensis* |
|  |  |  |  |  |  |  | *Phyllostachys edulis* |  |  |
|  |  |  |  |  |  |  | *Cunninghamia lanceola* |  |  |
| MPF | MPF-9 | 15.38 | 9.22 | 1680 | 0.74 | 35 | *Pinus massoniana* | *Loropetalum chinense* | *Loropetalum chinense* |
| MMF | MMF-10 | 14.43 | 9.04 | 1560 | 0.68 | 35 | *Pinus massoniana* | *Loropetalum chinense* | *Ophiopogon bodinieri* |
|  |  |  |  |  |  |  | *Angiospermae* |  |  |
|  |  |  |  |  |  |  | *Liquidambar formosana* |  |  |
| MPF | MPF-11 | 21.08 | 11.28 | 1260 | 0.55 | 35 | *Pinus massoniana* | *Camellia oleifera* | *Ophiopogon bodinieri* |
| MPF | MPF-12 | 15.88 | 8.98 | 1440 | 0.63 | 35 | *Pinus massoniana* | *Lindera glauca* | *Quercus aliena* |
| BMF | BMF-13 | 21.58 | 12.57 | 780 | 0.48 | 65 | *Cinnamomum camphora* | *Various species of scattered bamboo* | *Ardisia japonica* |
|  |  |  |  |  |  |  | *Liquidambar formosana* |  |  |
| BMF | BMF-14 | 28.44 | 14.81 | 660 | 0.41 | 65 | *Cinnamomum camphora* | *Various species of scattered bamboo* | *Ophiopogon bodinieri* |
|  |  |  |  |  |  |  | *Pinus elliottii* |  |  |
|  |  |  |  |  |  |  | *Liquidambar formosana* |  |  |
| CMF | CMF-15 | 21.11 | 12.14 | 1020 | 0.63 | 65 | *Pinus elliottii* | *Eurya japonica* | *Ardisia japonica* |
|  |  |  |  |  |  |  | *Cunninghamia lanceola* |  |  |
|  |  |  |  |  |  |  | *Cinnamomum camphora* |  |  |
| CMF | CMF-16 | 21.93 | 12.08 | 780 | 0.48 | 65 | *Cunninghamia lanceola* | *Various species of scattered bamboo* | *Eurya japonica* |
|  |  |  |  |  |  |  | *Pinus elliottii* |  |  |
|  |  |  |  |  |  |  | *Liquidambar formosana* |  |  |
| MPF | MPF-17 | 16.33 | 9.07 | 1380 | 0.67 | 35 | *Pinus massoniana* | *Camellia oleifera* | *Eurya japonica* |
| MMF | MMF-18 | 18.38 | 9.59 | 780 | 0.38 | 35 | *Pinus massoniana* | *Trachycarpus fortunei* | *Ophiopogon bodinieri* |
|  |  |  |  |  |  |  | *Liquidambar formosana* |  |  |
|  |  |  |  |  |  |  | *Phyllostachys edulis* |  |  |
| CBF | CBF-19 | 18.72 | 7.76 | 780 | 0.38 | 35 | *Liquidambar formosana* | *Quercus* | *Pteridium aquilinum* |
|  |  |  |  |  |  |  | *Pinus massoniana* |  |  |
|  |  |  |  |  |  |  | *Cunninghamia lanceola* |  |  |
| MPF | MPF-20 | 12.42 | 6.74 | 2400 | 1.00 | 35 | *Pinus massoniana* | *Camellia oleifera* | *Pteridium aquilinum* |
| BMF | BMF-21 | 16.87 | 10.15 | 900 | 0.59 | 30 | *Elaeocarpus decipiens* | *Other shrubs* | *Pteridium aquilinum* |
|  |  |  |  |  |  |  | *Cinnamomum camphora* |  |  |
|  |  |  |  |  |  |  | *Liquidambar formosana* |  |  |
| BMF | BMF-22 | 20.83 | 11.99 | 780 | 0.51 | 30 | *Liquidambar formosana* | *Other shrubs* | *Symplocos sumuntia* |
|  |  |  |  |  |  |  | *Diospyros kaki* |  |  |
|  |  |  |  |  |  |  | *Robinia pseudoacacia* |  |  |
| BMF | BMF-23 | 22.85 | 12.28 | 1500 | 0.80 | 30 | *Liquidambar formosana* | *Other shrubs* | *Pteridium aquilinum* |
|  |  |  |  |  |  |  | *Pinus elliottii* |  |  |
|  |  |  |  |  |  |  | *Quercus* |  |  |
| BMF | BMF-24 | 19.19 | 11.55 | 1800 | 0.81 | 30 | *Liquidambar formosana* | *Quercus* | *Castanopsis chinensis* |
|  |  |  |  |  |  |  | *Elaeocarpus decipiens* |  |  |
|  |  |  |  |  |  |  | *Pinus elliottii* |  |  |
| MMF | MMF-25 | 24.55 | 12.81 | 660 | 0.37 | 30 | *Pinus massoniana* | *Camellia oleifera* | *Cyclosorus interruptus* |
|  |  |  |  |  |  |  | *Elaeocarpus decipiens* |  |  |
|  |  |  |  |  |  |  | *Crataegus hupehensis* |  |  |
| LMF | LMF-26 | 20.08 | 11.67 | 1260 | 0.70 | 30 | *Cunninghamia lanceola* | *Camellia oleifera* | *Lindera aggregate* |
|  |  |  |  |  |  |  | *Liquidambar formosana* |  |  |
|  |  |  |  |  |  |  | *Pinus massoniana* |  |  |
| BMF | BMF-27 | 13.05 | 8.87 | 1680 | 0.72 | 30 | *Elaeocarpus decipiens* | *Camellia oleifera* | *Lindera aggregate* |
|  |  |  |  |  |  |  | *Liquidambar formosana* |  |  |
|  |  |  |  |  |  |  | *Cinnamomum camphora* |  |  |
| BMF | BMF-28 | 14.73 | 9.68 | 1560 | 0.70 | 30 | *Crataegus hupehensis* | *Camellia oleifera* | *Cyclosorus interruptus* |
|  |  |  |  |  |  |  | *Pinus massoniana* |  |  |
|  |  |  |  |  |  |  | *Liquidambar formosana* |  |  |
| EPF | EPF-29 | 16.16 | 7.87 | 900 | 0.43 | 25 | *Pinus elliottii* | *Vitex negundo* | *Oplismenus undulatifolius* |
| EPF | EPF-30 | 17.15 | 9.47 | 1260 | 0.60 | 25 | *Pinus elliottii* | *Bamboo Shrubland* | *Ophiopogon bodinieri* |
| EPF | EPF-31 | 20.94 | 10.85 | 900 | 0.43 | 25 | *Pinus elliottii* | *Vitex negundo* | *Ophiopogon bodinieri* |
| EPF | EPF-32 | 17.58 | 9.66 | 1140 | 0.54 | 25 | *Pinus elliottii* | *Vitex negundo* | *Ophiopogon bodinieri* |
| MMF | MMF-33 | 22.31 | 12.15 | 2220 | 0.87 | 25 | *Pinus massoniana* | *Eurya japonica* | *Cyclosorus interruptus* |
|  |  |  |  |  |  |  | *Camellia oleifera* |  |  |
|  |  |  |  |  |  |  | *Liquidambar formosana* |  |  |
| MPF | MPF-34 | 21.31 | 11.61 | 960 | 0.38 | 25 | *Pinus massoniana* | *Quercus* | *Eurya japonica* |
| MMF | MMF-35 | 14.94 | 9.13 | 2220 | 0.87 | 25 | *Pinus massoniana* | *Eurya japonica* | *Pteridium aquilinum* |
|  |  |  |  |  |  |  | *Liquidambar formosana* |  |  |
|  |  |  |  |  |  |  | *Pinus elliottii* |  |  |
| MPF | MPF-36 | 17.38 | 9.75 | 1200 | 0.47 | 25 | *Pinus massoniana* | *Eurya japonica* | *Pteridium aquilinum* |
| LMF | LMF-37 | 15.36 | 9.45 | 960 | 0.48 | 20 | *Cunninghamia lanceola* | *Camellia oleifera* | *Pteridium aquilinum* |
|  |  |  |  |  |  |  | *Liquidambar formosana* |  |  |
|  |  |  |  |  |  |  | *Pinus elliottii* |  |  |
| CMF | CMF-38 | 21.16 | 12.04 | 840 | 0.42 | 20 | *Pinus elliottii* | *Eurya japonica* | *Pteridium aquilinum* |
|  |  |  |  |  |  |  | *Cunninghamia lanceola* |  |  |
|  |  |  |  |  |  |  | *Pinus massoniana* |  |  |
| CMF | CMF-39 | 13.78 | 8.70 | 780 | 0.39 | 20 | *Cunninghamia lanceola* | *Camellia oleifera* | *Parnassia monochorifolia* |
|  |  |  |  |  |  |  | *Pinus elliottii* |  |  |
|  |  |  |  |  |  |  | *Liquidambar formosana* |  |  |
| CMF | CMF-40 | 16.87 | 10.53 | 1380 | 0.70 | 20 | *Pinus elliottii* | *Eurya japonica* | *Pteridium aquilinum* |
|  |  |  |  |  |  |  | *Liquidambar formosana* |  |  |
|  |  |  |  |  |  |  | *Cunninghamia lanceola* |  |  |
| LMF | LMF-41 | 16.06 | 10.81 | 1140 | 0.80 | 30 | *Cunninghamia lanceola* | *Camellia oleifera* | *Ficus fistulosa* |
|  |  |  |  |  |  |  | *Liquidambar formosana* |  |  |
|  |  |  |  |  |  |  | *Fagus longipetiolata* |  |  |
| MMF | MMF-42 | 19.70 | 11.17 | 1500 | 1.00 | 30 | *Pinus massoniana* | Other shrubs | *Lophatherum gracile* |
|  |  |  |  |  |  |  | *Cunninghamia lanceola* |  |  |
|  |  |  |  |  |  |  | *Cinnamomum camphora* |  |  |
| BMF | BMF-43 | 22.74 | 12.41 | 1140 | 0.80 | 30 | *Liquidambar formosana* | *Cinnamomum camphora* | *Cyclosorus interruptus* |
|  |  |  |  |  |  |  | *Cunninghamia lanceola* |  |  |
|  |  |  |  |  |  |  | Quercus |  |  |
| LMF | LMF-44 | 22.05 | 11.90 | 1080 | 0.76 | 30 | *Cunninghamia lanceola* | Other shrubs | *Clerodendrum cyrtophyllum* |
|  |  |  |  |  |  |  | Quercus |  |  |
|  |  |  |  |  |  |  | *Pinus massoniana* |  |  |
| MPF | MPF-45 | 17.27 | 9.79 | 900 | 0.57 | 20 | *Pinus massoniana* | *Loropetalum chinense* | *Loropetalum chinense* |
| MPF | MPF-46 | 17.54 | 10.06 | 1080 | 0.69 | 20 | *Pinus massoniana* | *Loropetalum chinense* | *Ophiopogon bodinieri* |
| MPF | MPF-47 | 17.93 | 10.22 | 1140 | 0.72 | 20 | *Pinus massoniana* | *Loropetalum chinense* | *Ophiopogon bodinieri* |
| CBF | CBF-48 | 21.16 | 11.59 | 660 | 0.42 | 20 | *Liquidambar formosana* | *Loropetalum chinense* | *Miscanthus sinensis* |
|  |  |  |  |  |  |  | *Pinus elliottii* |  |  |
|  |  |  |  |  |  |  | *Pinus massoniana* |  |  |
| CPF | CPF-49 | 16.45 | 10.02 | 1095 | 0.65 | 35 | *Cunninghamia lanceola* | Other shrubs | *Parnassia monochorifolia* |
| SF | SF-50 | 2.50 | 3.78 | 26000* | - | 55 | *Loropetalum chinense* | *Loropetalum chinense* | *Ophiopogon bodinieri* |
|  |  |  |  |  |  |  | *Lindera aggregate* |  |  |
|  |  |  |  |  |  |  | *Robinia pseudoacacia* |  |  |
| SF | SF-51 | 2.44 | 3.49 | 14000* | - | 55 | *Loropetalum chinense* | *Loropetalum chinense* | *Ophiopogon bodinieri* |
|  |  |  |  |  |  |  | *Robinia pseudoacacia* |  |  |
|  |  |  |  |  |  |  | *Quercus aliena* |  |  |
| SF | SF-52 | 2.89 | 5.37 | 16800* | - | 55 | *Loropetalum chinense* | *Loropetalum chinense* | *Ophiopogon bodinieri* |
|  |  |  |  |  |  |  | *Robinia pseudoacacia* |  |  |
|  |  |  |  |  |  |  | *Symplocos sumuntia* |  |  |
| LMF | LMF-53 | 20.35 | 11.69 | 720 | 0.54 | 30 | *Cunninghamia lanceola* | *Diospyros kaki* | *Pteridium aquilinum* |
|  |  |  |  |  |  |  | *Liquidambar formosana* |  |  |
|  |  |  |  |  |  |  | *Camellia oleifera* |  |  |
| CPF | CPF-54 | 20.10 | 11.76 | 1020 | 0.76 | 30 | *Cunninghamia lanceola* | *Loropetalum chinense* | *Pteridium aquilinum* |
| CPF | CPF-55 | 18.25 | 11.52 | 1440 | 1.00 | 30 | *Cunninghamia lanceola* | Other shrubs | *Pteridium aquilinum* |
| CPF | CPF-56 | 19.29 | 11.25 | 1920 | 1.00 | 30 | *Cunninghamia lanceola* | Other shrubs | *Pteridium aquilinum* |
| CMF | CMF-57 | 13.64 | 8.59 | 1200 | 0.44 | 20 | *Pinus elliottii* | *Loropetalum chinense* | *Loropetalum chinense* |
|  |  |  |  |  |  |  | *Cunninghamia lanceola* |  |  |
|  |  |  |  |  |  |  | *Elaeocarpus decipiens* |  |  |
| CPF | CPF-58 | 12.41 | 8.15 | 1020 | 0.38 | 20 | *Cunninghamia lanceola* | *Rhus chinensis* | *Camellia oleifera* |
| CMF | CMF-59 | 14.92 | 9.14 | 1860 | 0.69 | 20 | *Cunninghamia lanceola* | *Rhus chinensis* | *Lindera aggregate* |
|  |  |  |  |  |  |  | *Pinus elliottii* |  |  |
|  |  |  |  |  |  |  | *Liquidambar formosana* |  |  |
| CMF | CMF-60 | 12.10 | 7.75 | 1320 | 0.49 | 20 | *Cunninghamia lanceola* | *Loropetalum chinense* | *Miscanthus sinensis* |
|  |  |  |  |  |  |  | *Pinus elliottii* |  |  |
|  |  |  |  |  |  |  | *Liquidambar formosana* |  |  |
| EPF | EPF-61 | 20.06 | 11.69 | 1200 | 0.83 | 20 | *Pinus elliottii* | *Liquidambar formosana* | *Cyclosorus interruptus* |
| EPF | EPF-62 | 18.66 | 11.42 | 1500 | 1.00 | 20 | *Pinus elliottii* | *Liquidambar formosana* | *Gynostemma pentaphyllum* |
| EPF | EPF-63 | 17.64 | 9.95 | 1020 | 0.70 | 20 | *Pinus elliottii* | *Liquidambar formosana* | *Cyclosorus interruptus* |
| EPF | EPF-64 | 20.03 | 10.99 | 1200 | 0.83 | 20 | *Pinus elliottii* | *Liquidambar formosana* | *Liquidambar formosana* |
| BMF | BMF-65 | 16.24 | 10.01 | 2460 | 0.89 | 50 | *Elaeocarpus decipiens* | Other shrubs | *Cyclosorus interruptus* |
|  |  |  |  |  |  |  | *Fagus longipetiolata* |  |  |
|  |  |  |  |  |  |  | *Cinnamomum camphora* |  |  |
| MPF | MPF-66 | 18.17 | 10.31 | 3060 | 1.00 | 50 | *Pinus massoniana* | *Loropetalum chinense* | *Lindera aggregate* |
| MMF | MMF-67 | 14.32 | 8.76 | 3000 | 1.00 | 50 | *Pinus massoniana* | Other shrubs | *Bambusoideae (Young bamboo)* |
|  |  |  |  |  |  |  | *Fagus longipetiolata* |  |  |
|  |  |  |  |  |  |  | *Cinnamomum camphora* |  |  |
| BMF | BMF-68 | 19.15 | 11.06 | 1980 | 0.72 | 50 | *Fagus longipetiolata* | Other shrubs | *Lindera aggregate* |
|  |  |  |  |  |  |  | *Pinus massoniana* |  |  |
|  |  |  |  |  |  |  | *Terminalia neotaliala* |  |  |
| BMF | BMF-69 | 11.72 | 8.15 | 840 | 0.64 | 25 | *Liquidambar formosana* | *Eurya japonica* | *Pteridium aquilinum* |
|  |  |  |  |  |  |  | *Pinus massoniana* |  |  |
|  |  |  |  |  |  |  | *Quercus* |  |  |
| MPF | MPF-70 | 20.95 | 12.00 | 1260 | 0.96 | 25 | *Pinus massoniana* | Dicotyledon | *Dendropanax dentiger* |
| MPF | MPF-71 | 22.42 | 12.26 | 1380 | 1.00 | 25 | *Pinus massoniana* | Dicotyledon | *Syzygium buxifolium* |
| CMF | CMF-72 | 9.34 | 6.56 | 1320 | 1.00 | 25 | *Cunninghamia lanceola* | Dicotyledon | *Cunninghamia lanceolata* |
|  |  |  |  |  |  |  | *Pinus massoniana* |  |  |
|  |  |  |  |  |  |  | *Diospyros kaki* |  |  |
| MPF | MPF-73 | 18.81 | 10.67 | 2160 | 0.65 | 35 | *Pinus massoniana* | *Camellia oleifera* | *Pteridium aquilinum* |
| CBF | CBF-74 | 21.33 | 11.30 | 2940 | 0.89 | 35 | *Paulownia fortunei* | *Cinnamomum camphora* | *Cunninghamia lanceolata* |
|  |  |  |  |  |  |  | *Camellia oleifera* |  |  |
|  |  |  |  |  |  |  | *Pinus massoniana* |  |  |
| MPF | MPF-75 | 20.77 | 11.36 | 960 | 0.29 | 35 | *Pinus massoniana* | Viburnum | *Pteridium aquilinum* |
| MPF | MPF-76 | 18.71 | 10.52 | 2040 | 0.61 | 35 | *Pinus massoniana* | *Cinnamomum camphora* | *Pteridium aquilinum* |
| EPF | EPF-77 | 13.89 | 8.36 | 2160 | 0.77 | 20 | *Pinus elliottii* | *Citrus maxima* | *Pteridium aquilinum* |
| EPF | EPF-78 | 16.58 | 9.42 | 1680 | 0.60 | 20 | *Pinus elliottii* | *Camellia oleifera* | *Rhynchosia volubilis* |
| EPF | EPF-79 | 12.56 | 7.72 | 2340 | 0.83 | 20 | *Pinus elliottii* | *Loropetalum chinense* | *Loropetalum chinense* |
| CMF | CMF-80 | 12.42 | 7.42 | 2820 | 1.00 | 20 | *Pinus massoniana* | *Camellia oleifera* | *Loropetalum chinense* |
|  |  |  |  |  |  |  | *Pinus elliottii* |  |  |
|  |  |  |  |  |  |  | *Cunninghamia lanceola* |  |  |
| FPF | FPF-81 | 17.27 | 10.33 | 960 | 0.75 | 25 | *Liquidambar formosana* | *Cinnamomum camphora* | *Cyperus rotundus* |
| EPF | EPF-82 | 18.52 | 11.84 | 780 | 0.61 | 25 | *Pinus elliottii* | Other shrubs | *Pteridium aquilinum* |
| EPF | EPF-83 | 27.22 | 13.75 | 540 | 0.55 | 25 | *Pinus elliottii* | Other shrubs | *Pteridium aquilinum* |
| EPF | EPF-84 | 20.29 | 12.40 | 540 | 0.55 | 25 | *Pinus elliottii* | Other shrubs | *Miscanthus sinensis* |
| CPF | CPF-85 | 14.64 | 9.19 | 2700 | 0.91 | 35 | *Cunninghamia lanceola* | *Lindera reflexa* | *Cyclosorus interruptus* |
| FPF | FPF-86 | 24.51 | 12.94 | 780 | 0.85 | 35 | *Liquidambar formosana* | *Cinnamomum camphora* | *Parnassia monochorifolia* |
| MMF | MMF-87 | 20.64 | 12.12 | 960 | 0.95 | 35 | *Pinus massoniana* | *Cinnamomum camphora* | *Lindera aggregate* |
|  |  |  |  |  |  |  | *Liquidambar formosana* |  |  |
|  |  |  |  |  |  |  | *Castanopsis sclerophylla* |  |  |
| FPF | FPF-88 | 21.81 | 12.35 | 540 | 0.83 | 35 | *Liquidambar formosana* | *Cinnamomum camphora* | *Pteridium aquilinum* |
| MPF | MPF-89 | 18.26 | 10.41 | 900 | 0.96 | 35 | *Pinus massoniana* | *Cinnamomum camphora* | *Lindera aggregate* |
| CPF | CPF-90 | 20.24 | 11.29 | 660 | 0.31 | 50 | *Cunninghamia lanceola* | Other shrubs | *Pteridium aquilinum* |
| CPF | CPF-91 | 20.18 | 11.47 | 960 | 0.45 | 50 | *Cunninghamia lanceola* | *Rhododendron simsii* | *Pteridium aquilinum* |
| BMF | BMF-92 | 22.27 | 12.06 | 300 | 0.20 | 50 | Quercus | *Rhododendron simsii* | *Parnassia monochorifolia* |
| CPF | CPF-93 | 21.23 | 12.01 | 1500 | 0.70 | 50 | *Cunninghamia lanceola* | *Lindera reflexa* | *Parnassia monochorifolia* |
| SG | SG-94 | 1.10 | 0.80 | 400* | - | - | *-* | *Liquidambar formosana* | *Imperata cylindrica* |
| SG | SG-95 | 1.10 | 0.90 | 400* | - | - | *-* | *Liquidambar formosana* | *Imperata cylindrica* |
| SG | SG-96 | 1.30 | 1.35 | 800* | - | - | *-* | *Liquidambar formosana* | *Imperata cylindrica* |
| BMF | BMF-97 | 13.50 | 13.55 | 1313 | 0.81 | 30 | *Liquidambar formosana* | *Other shrubs* | *Pteridium aquilinum* |
|  |  |  |  |  |  |  | *Angiospermae* |  |  |
| MMF | MMF-98 | 24.34 | 12.46 | 457 | 0.65 | 20 | *Pinus massoniana* | *Eurya japonica* | *Pteridium aquilinum* |
|  |  |  |  |  |  |  | *Cinnamomum camphora* |  |  |
| MPF | MPF-99 | 21.48 | 12.39 | 766 | 0.55 | 30 | *Pinus massoniana* | *Camellia oleifera* | *Pteridium aquilinum* |
| CPF | CPF-100 | 17.20 | 13.04 | 1775 | 0.75 | 25 | *Cunninghamia lanceola* | *Other shrubs* | *Pteridium aquilinum* |
| MMF | MMF-101 | 23.16 | 13.70 | 493 | 0.61 | 30 | *Pinus massoniana* | *Eurya japonica* | *Piper hancei* |
|  |  |  |  |  |  |  | *Cinnamomum camphora* |  |  |
|  |  |  |  |  |  |  | *Liquidambar formosana* |  |  |
| MMF | MMF-102 | 20.42 | 11.58 | 580 | 0.55 | 25 | *Pinus massoniana* | *Other shrubs* | *Tetrastigma obtectum* |
|  |  |  |  |  |  |  | *Cinnamomum camphora* |  |  |
|  |  |  |  |  |  |  | *Liquidambar formosana* |  |  |
| MMF | MMF-103 | 20.62 | 13.10 | 591 | 0.45 | 30 | *Pinus massoniana* | Other shrubs | *Pteridium aquilinum* |
|  |  |  |  |  |  |  | *Liquidambar formosana* |  |  |
|  |  |  |  |  |  |  | *Cinnamomum camphora* |  |  |
| CPF | CPF-104 | 18.09 | 10.97 | 591 | 0.45 | 36 | *Cunninghamia lanceola* | Other shrubs | *Pteridium aquilinum* |

**Note:** CPF: *Cunninghamia lanceolata* (Lamb.) Hook pure forest, MPF: *Pinus massoniana* pure forest, EPF: *Pinus elliottii* Pure forest, FPF: *Liquidambar formosana* Hance Pure forest, HPF: P*hyllostachys heterocycla* (Carr.) Mitford cv. Pubescens Pure forest, CMF: Coniferous mixed forest, BMF: Broad-leaved mixed forest, CBF: Coniferous Broad-leaved mixed forest, LMF: *Cunninghamia lanceolata* (Lamb.) Hook coniferous and broad-leaved mixed forest, MMF: P*inus massoniana* broadleaf-conifer mixed forest, SF: Broad-leaved shrub forest, SG: Broad-leaved shrub grass, DBH: Mean basal diameter, H: Mean tree height, Den: Density, DCC: degree of canopy closure, *: indicates the number of shrubs with a diameter at 30cm height greater than 1cm.

**Table S3.** The quantitative indicators of flammability characteristics of the 39 plant families.

| Family | N.Spe | N.Sam | IP (℃) | CD (s) | CV (KJ/g) | AC (％) | DT (h) |
| --- | --- | --- | --- | --- | --- | --- | --- |
| Herbs | 32 | 779 |  |  |  |  |  |
| Araliaceae | 1 | 1 | 242±2.2ab | 58.5±4.47a | 17.564±1.14bc | 4.9±0bc | 39±0ab |
| Arecaceae | 1 | 13 | 232.8±0bc | 55.8±0a | 17.131±0.73bc | 7.3±2.1abc | 30±0b |
| Asparagaceae | 1 | 11 | 236±0bc | 60.9±0a | 17.572±0.49bc | 7.8±2abc | 38.7±2.1ab |
| Caryophyllaceae | 1 | 1 | 232.8±0bc | 55.8±3.66a | 17.131±0bc | 7.3±0abc | 30±0b |
| Celastraceae | 1 | 5 | 236.3±0bc | 59.1±2.83a | 17.802±0.49bc | 6.2±1.7abc | 55.8±0a |
| Cucurbitaceae | 1 | 1 | 239.8±0ab | 64±0a | 16.236±0.63c | 9.5±0ab | 24±0b |
| Cupressaceae | 1 | 24 | 236.7±0bc | 57.7±0a | 17.204±0bc | 7.5±0.9abc | 37±0ab |
| Cyperaceae | 1 | 1 | 239±2.79abc | 57.9±0.24a | 16.816±0c | 10.1±2.2ab | 63±0a |
| Dennstaedtiaceae | 1 | 30 | 237.3±6.14bc | 61±2.91a | 17.548±0.44bc | 6.9±0abc | 42.9±0ab |
| Fabaceae | 1 | 13 | 238.7±2.85abc | 57.3±0a | 17.935±0abc | 5.2±0bc | 30±0b |
| Fagaceae | 2 | 49 | 238.1±3.85abc | 59.6±2.93a | 18.404±0ab | 5.4±0bc | 37.5±8ab |
| Hamamelidaceae | 1 | 138 | 239.3±0ab | 60.8±0a | 17.908±0.41abc | 5.9±2.9bc | 31.5±10.4b |
| Juncaceae | 1 | 2 | 233.8±0bc | 57.1±0a | 17.407±0bc | 7.5±0abc | 42±16.6ab |
| Lamiaceae | 2 | 2 | 235.6±4.24bc | 58.9±0a | 17.444±0bc | 9±1.7ab | 33±7.5b |
| Lauraceae | 1 | 141 | 242.7±0a | 61.6±0a | 17.611±0bc | 7.2±2.4abc | 36±4.2b |
| Moraceae | 1 | 37 | 238.3±2.95abc | 62.1±1.42a | 17.756±0bc | 10.7±0a | 36±9.2b |
| Myrtaceae | 1 | 1 | 242±0ab | 58.5±3.44a | 17.564±0bc | 4.9±0bc | 39±15.9ab |
| Pentaphylacaceae | 1 | 72 | 235.6±0bc | 58.1±0a | 17.464±0.92bc | 6.9±0abc | 49±10.7ab |
| Piperaceae | 1 | 2 | 238.2±0abc | 59.9±0a | 17.634±0.72bc | 7±1.7abc | 33±4.2b |
| Poaceae | 6 | 49 | 238.8±0abc | 60±6.28a | 17.311±0.82bc | 7.9±2.5ab | 39.2±6.2ab |
| Polygonaceae | 1 | 1 | 236.2±4.11bc | 56.8±0a | 16.753±0c | 10.3±0ab | 36±0b |
| Primulaceae | 1 | 2 | 231±5.1c | 58.3±0a | 17.89±0abc | 7.8±0abc | 42±0ab |
| Symplocaceae | 1 | 28 | 240.1±2.57ab | 57.5±2.75a | 18.06±0.67abc | 6±0abc | 39±0ab |
| Theaceae | 1 | 148 | 244.8±0a | 62.5±0a | 19.445±0a | 3.5±1.2c | 24±11.3b |
| Thelypteridaceae | 1 | 7 | 239.6±4.63ab | 60.9±0.73a | 16.823±0c | 8.7±0ab | 33.9±0b |
| Shurbs | 37 | 921 |  |  |  |  |  |
| Anacardiaceae | 1 | 20 | 232.5±0def | 58.1±3bcd | 18.489±0ab | 4.1±0bc | 100.8±12.6b |
| Aquifoliaceae | 1 | 20 | 224.6±0.68g | 54.3±0.18d | 18.149±0abc | 3.7±3bc | 75.6±30.1cde |
| Arecaceae | 1 | 13 | 220.8±0h | 53.8±0d | 16.375±0d | 2.6±0c | 147.8±10.8a |
| Cornaceae | 1 | 3 | 233.5±3.66cdef | 64.5±0.41ab | 17.184±0.28cd | 3.1±0.7bc | 37±23.9f |
| Cupressaceae | 1 | 24 | 245.2±0.69a | 59.5±1.9bc | 18.119±2.86abc | 3.6±0bc | 40.5±0f |
| Ebenaceae | 1 | 16 | 233±2.7cdef | 57.6±0bcd | 18.452±0.32ab | 2.7±2c | 59.8±22.7def |
| Elaeagnaceae | 1 | 12 | 238.7±0.57b | 57.3±2.03bcd | 18.726±0.44ab | 2.6±0.9c | 33.6±0f |
| Ericaceae | 2 | 15 | 233.5±2.7cdef | 58.7±4.83bc | 18.746±0.63a | 2.7±2.7c | 75.9±16.4cde |
| Euphorbiaceae | 1 | 12 | 235.9±2.43bc | 57.7±8.2bcd | 17.53±0bc | 5±0ab | 37±11.9f |
| Fabaceae | 1 | 13 | 231.9±1.14ef | 58.5±1.66bcd | 17.698±0.15bc | 5.9±1.2a | 37±0f |
| Fagaceae | 2 | 49 | 238±0b | 61.1±2.08b | 16.539±0.19d | 3.3±0.6bc | 97.2±0b |
| Hamamelidaceae | 2 | 138 | 232.9±1.79def | 59.2±2.85bc | 17.462±0.05c | 3.8±0.5bc | 79.9±0cd |
| Lauraceae | 5 | 141 | 235.8±0bc | 60.7±0b | 18.712±0ab | 3.6±1.2bc | 67.1±46.8de |
| Moraceae | 2 | 37 | 234.6±1.42c | 58.7±4.84bc | 17.908±0.2abc | 3.9±1.9bc | 81.5±11.8bc |
| Pentaphylacaceae | 1 | 72 | 234.3±0cd | 62.6±0ab | 18.216±0abc | 4.2±0b | 79±0cd |
| Poaceae | 1 | 49 | 226.2±0.94g | 60.7±0.02b | 18.369±0ab | 4.6±0ab | 90.7±0bc |
| Rosaceae | 1 | 4 | 231.9±0ef | 55.6±5.2cd | 17.389±0.58cd | 4.9±0ab | 50.4±1.8def |
| Rubiaceae | 2 | 24 | 231.9±5.28ef | 58.6±4.14bc | 17.436±0.77c | 4.4±0.3ab | 38.6±32.3f |
| Rutaceae | 2 | 24 | 231.8±2.88ef | 65.3±2.51a | 17.442±0c | 4.7±0.2ab | 57.1±0def |
| Scrophulariaceae | 1 | 12 | 238±0b | 62.1±0ab | 18.074±0.95abc | 4±1bc | 47±18ef |
| Symplocaceae | 1 | 28 | 237.8±1.52b | 58.3±0bcd | 17.494±0.08c | 3.7±0.8bc | 77.3±0cd |
| Theaceae | 1 | 148 | 233.5±2.99cdef | 62±1.01b | 18.37±0.9ab | 4±0.6bc | 63.2±13.5de |
| Verbenaceae | 3 | 32 | 234.1±0cde | 60.2±0b | 17.817±0.2bc | 5.8±1.3a | 79.5±3cd |
| Viburnaceae | 1 | 11 | 229.7±2.7f | 55.1±3.78cd | 18.022±0.78abc | 4.9±1.4ab | 70.6±22cde |
| Vitaceae | 1 | 4 | 233.2±6.59cdef | 60±0bc | 18.322±0.32abc | 3.1±0bc | 80.6±22.6bcd |

**Note:** The total number of plant families is 39, with 25 families in the shrub layer and 25 families in the herb layer, including an overlap of 11 families between them. N. Spe is number of species, N.Sam is number of samples, IP is ignition point, CD is combustion duration, CV is calorific value, AC is ash content, and DT is drying time. The identical letters in the same column indicated that they had no significance between different families in shrub or between different families in herbs.

**Supplementary Figures**


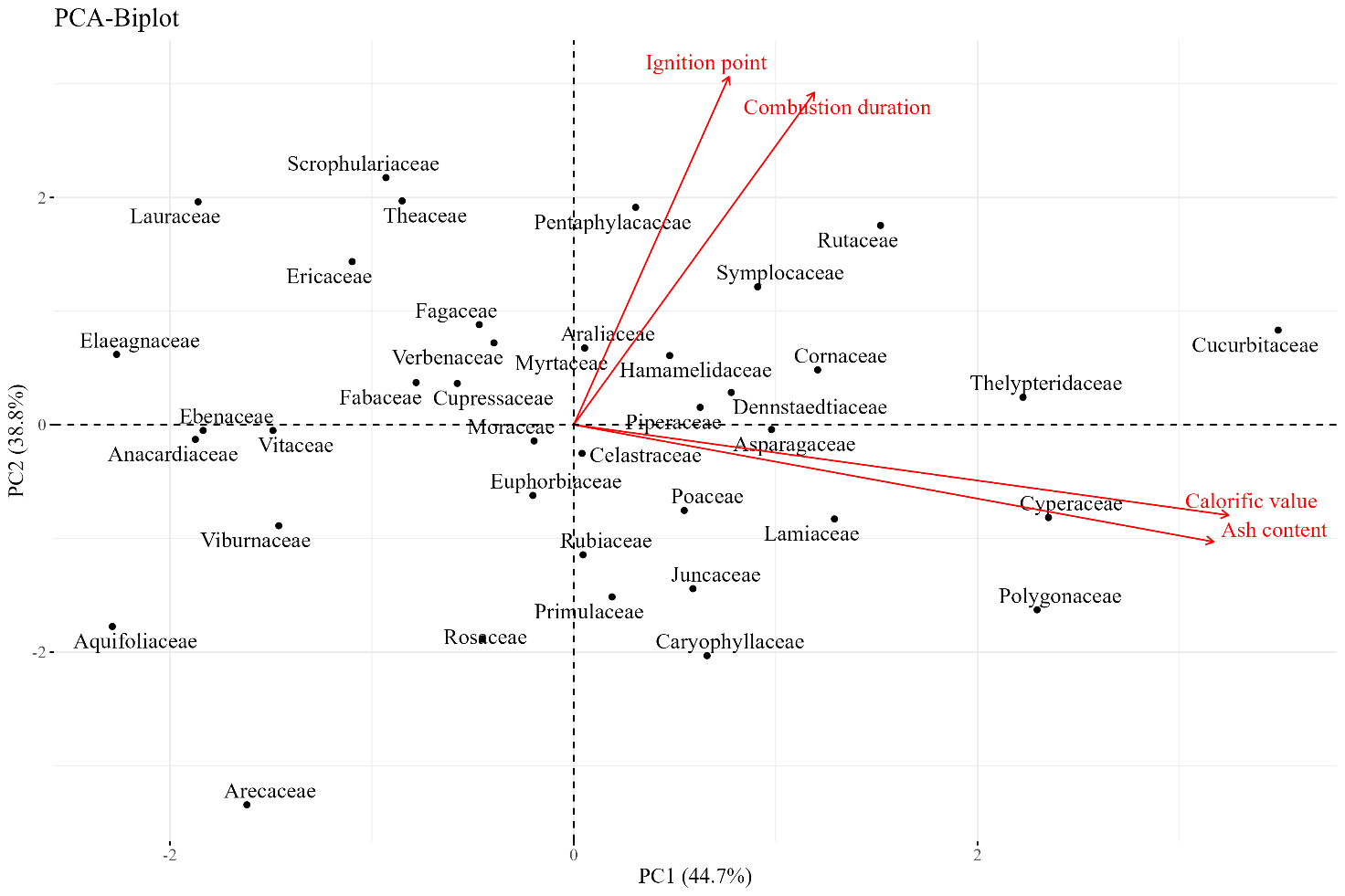


**Figure S1**. Principal component analysis (PCA) of the four measured flammability variables (IP: ignition point, CV: calorific value, AC: ash content, CD: combustion duration) showing the first two components PC1 and PC2 that explained a total of 83.5 % variation in the data. Each point represents the average score of a combustible component.


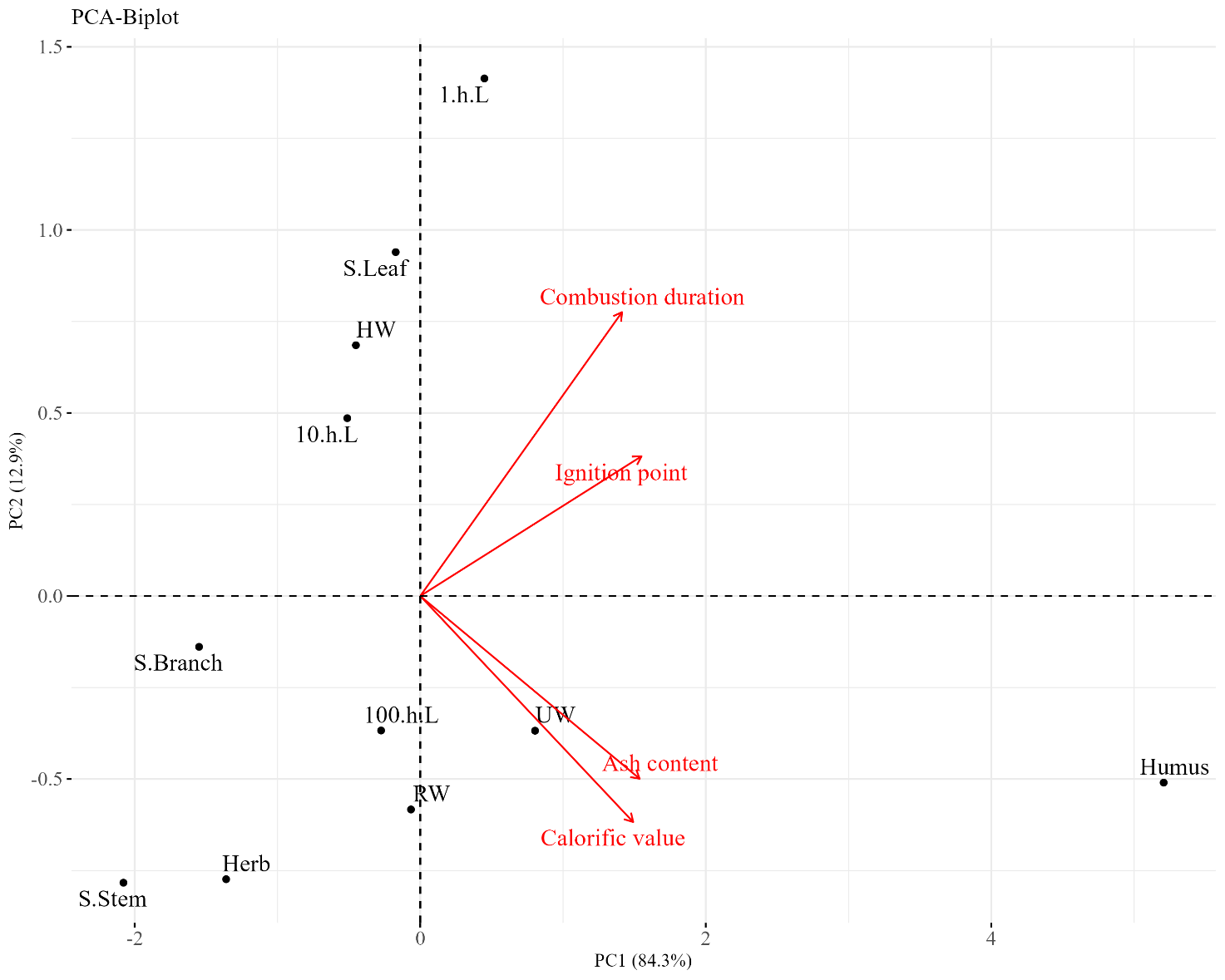


**Figure S2**. Principal component analysis (PCA) of the four measured flammability variables (IP: ignition point, CV: calorific value, AC: ash content, CD: combustion duration) showing the first two components PC1 and PC2 that explained a total of 97.2 % variation in the data. Each point represents the average score of a combustible component.


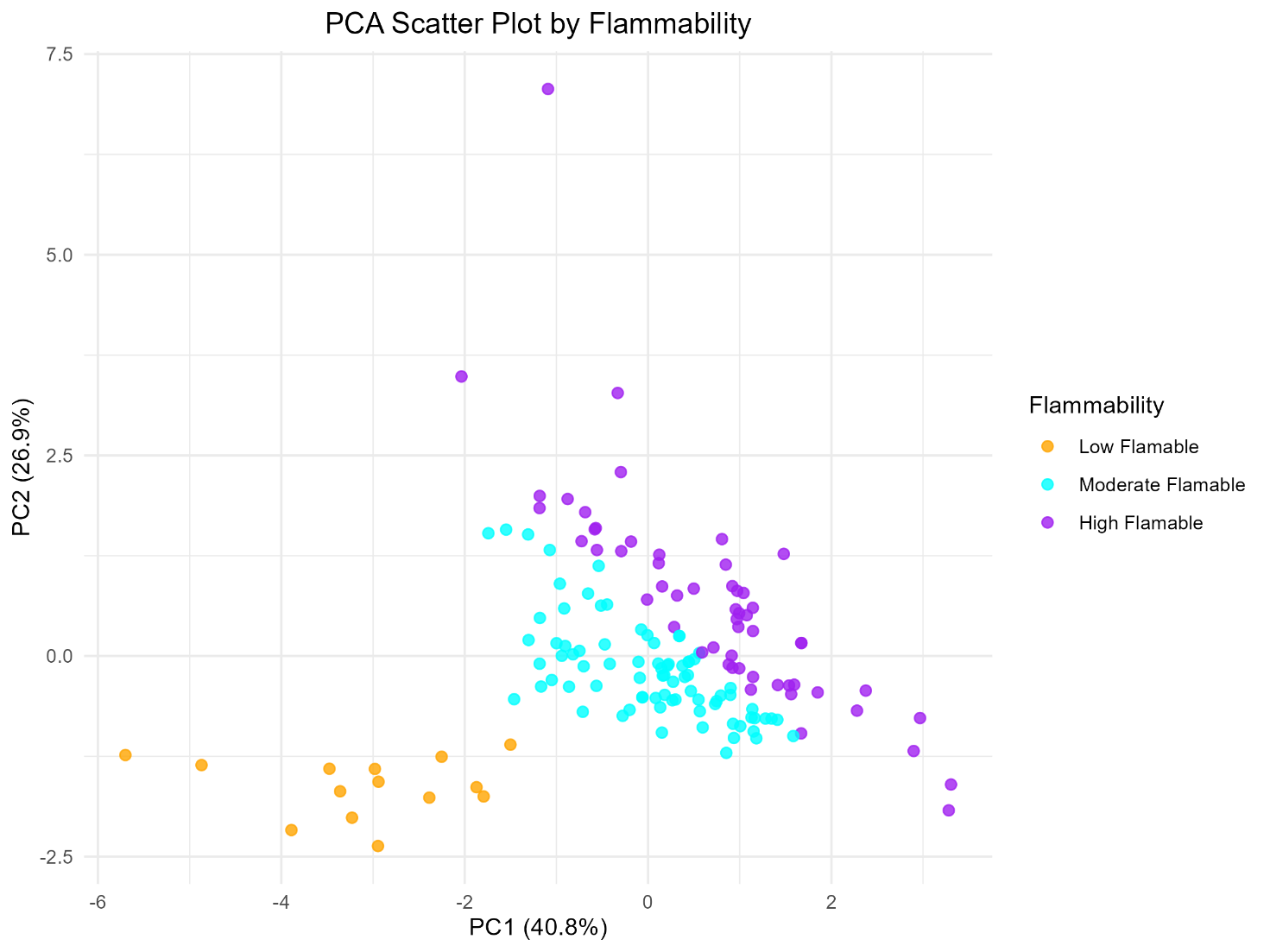


**Figure S3**. PCA of the average values of five recorded flammability traits (fuel moisture content (FM)、drying time (DT)、combustion duration (CD)、calorific value (CV)、ignition point (IP)) for each fuel type. The points represent species (n = 150). The color of the points indicates the flammability category, classified using k-means clustering.
